# PTPN2 inhibition unleashes response to STING agonism in head and neck squamous cell cancer

**DOI:** 10.1038/s41467-026-72372-1

**Published:** 2026-05-02

**Authors:** Zehua Li, Cong Fu, Kartik Sehgal, Ann Marie Egloff, Tran C. Thai, Omar Avila Monge, Cun Lan Chuong, Yaiza Senent, Maia Shea Lineberry, Benjamin K. Eschle, Koji Haratani, Elena V. Ivanova, Marco Campisi, Navin R. Mahadevan, John D. Quadarella, Patrick C. Gedeon, Jonathan D. Schoenfeld, Michihisa Kono, Caroline G. Fahey, Chayapatou Chayawatto, Kenneth Ngo, Cloud P. Paweletz, Prafulla C. Gokhale, Robert T. Manguso, Ravindra Uppaluri, David A. Barbie

**Affiliations:** 1 Department of Medical Oncology, Dana-Farber Cancer Institute, Boston, MA USA; 2 Harvard Medical School, Boston, MA USA; 3 Department of Surgery, Brigham and Women’s Hospital, Boston, MA USA; 4 Broad Institute of MIT and Harvard, Cambridge, MA USA; 5 Center for Cancer Research and Department of Medicine, Massachusetts General Hospital, Boston, MA USA; 6 Belfer Center for Applied Cancer Science, Dana-Farber Cancer Institute, Boston, MA USA; 7 Department of Pathology, Brigham and Women’s Hospital, Boston, MA USA; 8 Department of Pathology, University of Michigan, Ann Arbor, MI USA; 9 Lowe Center for Thoracic Oncology, Dana-Farber Cancer Institute, Boston, MA USA; 10 Parker Institute for Cancer Immunotherapy at Dana-Farber Cancer Institute, Boston, MA USA

**Keywords:** Tumour immunology, Tumour-suppressor proteins, Innate immunity, Tumour immunology, Cell death and immune response

## Abstract

cGAS-STING signaling can promote antitumor immunity, and tumor cell STING is suppressed in a variety of cancer subtypes that resist immune checkpoint blockade. Although STING agonists have failed clinical trials, precision approaches targeting restoration of tumor cell STING expression have yet to be explored. Here, we report that head and neck squamous cell cancer (HNSCC) exhibits a mechanism of STING suppression related to upregulation of protein tyrosine phosphatase non-receptor (PTPN) type 2 (PTPN2) that is also evident in other cancers. PTPN2 inhibition (PTPN2i) increases HNSCC tumor cell STING by restoring IFNγ-STAT1–mediated induction of STING mRNA. This restores sensitivity to STING agonism and natural killer cell activation, suppressing tumor growth in an immune cell-dependent manner in anti-PD-1 refractory syngeneic HNSCC mouse tumor models in female mice. Together, these findings demonstrate that PTPN2i can unleash STING agonist response, providing a rationale for the evaluation of this therapeutic combination in HNSCC and potentially other cancer types.

## Abstract (web summary)

STING agonism can promote antitumor immunity, however, mechanisms of resistance to STING activation have been described in several cancer types. Here the authors report that upregulation of PTPN2 in head and neck squamous cell carcinoma is associated with STING suppression, showing that PTPN2 inhibition restores tumor cell STING expression, and enhances response to STING agonism and antitumor immunity.

## Introduction

The cyclic GMP–AMP synthase (cGAS)–stimulator of interferon genes (STING) signaling pathway has emerged as a key regulator of innate immunity across multiple types of cancers^1,2^. cGAS recognizes cytosolic double-stranded DNA derived from microbial pathogens or aberrantly released self-DNA, and then catalyzes the production of the second messenger 2′3′-cyclic GMP-AMP (2′3′-cGAMP)^3^. 2′3′-cGAMP binds and activates STING, which subsequently activates TANK-binding kinase 1 (TBK1) to phosphorylate IRF3 (p-IRF3), resulting in the production of type I interferons, proinflammatory cytokines, and chemokines^2^. Pharmacologic activation of the cGAS-STING pathway by natural STING ligand cyclic di-nucleotides (CDNs), or synthetic STING agonists such as ADU-S100 (ADU), can promote antitumor immunity in the syngeneic mouse models, including head and neck squamous cell carcinoma (HNSCC)^4,5^. However, STING agonists have yet to achieve success in clinical trials, even when combined with anti-PD-1 therapy^6,7^. Potential underlying mechanisms for lack of efficacy may include suboptimal pharmacokinetics related to different STING agonist delivery methods, low tumor cell membrane permeability, as well as cytotoxicity of STING agonists to T cells^8–11^. Despite unresponsiveness to STING agonists likely due to suppression of STING expression in multiple cancer types^12^, clinical trials have yet to employ biomarker selection in further evaluation of STING agonists alone or in combination with other therapies.

Activation of tumor-intrinsic STING signaling stimulates immunogenic cell death and promotes tumor cell immunogenicity^13–17^, and overcomes immune checkpoint blockade (ICB) resistance in mouse models^18,19^. For example, restoring STING expression in immunotherapy-resistant KRAS-STK11/LKB1 mutant models of non-small cell lung cancer (NSCLC) and small cell lung cancer (SCLC) primes response to STING agonism^14,18,20,21^. Reversal of epigenetic STING silencing in B16F10 melanoma also recently demonstrated the importance of tumor cell STING expression relative to myeloid STING in mediating tumor immunogenicity^22^. In support of these findings, enforced expression of a gain-of-function STING mutant in colorectal cancer models was required to induce cancer cell-intrinsic interferon signaling and to sensitize resistant tumors to ICB therapy in vivo^19^, highlighting the critical role of tumor cell STING induction and activation of anti-tumor immunity.

HNSCC patients with low STING expression is associated with shorter progression-free survival with standard of care therapy^16^. Furthermore, mouse oral squamous cell carcinoma (MOC1) and MOC2 cells exhibited low STING expression relative to multiple immune cell populations and exhibited impaired response to CDNs^4,12,23^. Prior work also suggested that CDN-induced TNF-α and IFN-β were mainly derived from myeloid cell populations in syngeneic mouse tumors^4,12^, though systematic comparison of STING levels or responsiveness in HNSCC relative to other tumor cell lines was not performed. Recent work has also confirmed suppression of STING expression in multiple human HNSCC cell lines, as well as lack of response to the STING agonist ADU-S100 as compared with THP1 myeloid cells^24^. This result was similar in HPV positive cells as well as HPV negative cells, regardless of STING levels. However, once again, direct comparison with other known STING agonist-responsive human cancer cell lines was not performed, and the underlying mechanism that mediates suppression of STING signaling in HNSCC cells has been relatively underexplored.

Several mechanisms have been shown to suppress STING activation in tumor cells. Tumor cell STING mRNA expression can be epigenetically silenced by DNA methyltransferases (DNMTs) and the enhancer of zeste homolog 2 (EZH2) through hypermethylation^14^. However, these drugs can be cytotoxic, and we cannot fully rule out the impact of epigenetic silencing. Induction of three-prime repair exonuclease 1 (TREX1) can repress tumor cell STING-IFN signaling by degrading cytosolic dsDNA upstream, limiting STAT1-induced STING expression^18^. These findings suggest that additional mechanisms could be operative in cancer cells to inhibit STAT1-mediated transcriptional induction of STING. Recently, PTPN2 has been identified as a master regulator that inhibits antitumor immunity by negatively regulating IFN-β and IFN-γ signaling through dephosphorylation of JAK-STAT^25^. The PTPN2/PTPN1 inhibitor AC484 has been shown to promote antitumor immunity by enhancing NK cell and CD8+ Tcell function through JAK-STAT signaling^26^. However, whether PTPN2 might also regulate tumor STING expression in HNSCC and other cancer types has remained unexplored.

In this work, we identify PTPN2 upregulation as dominant mechanism of STING transcriptional silencing in HNSCC via impaired STAT1 activation. Therapeutically targeting PTPN2 with AC484 thus increases IFNγ-driven tumor cell STING expression and restores STING agonist response in vitro and in vivo.

## Results

### HNSCC cell lines exhibit reduced responsiveness to STING agonism

**Fig. 1:**
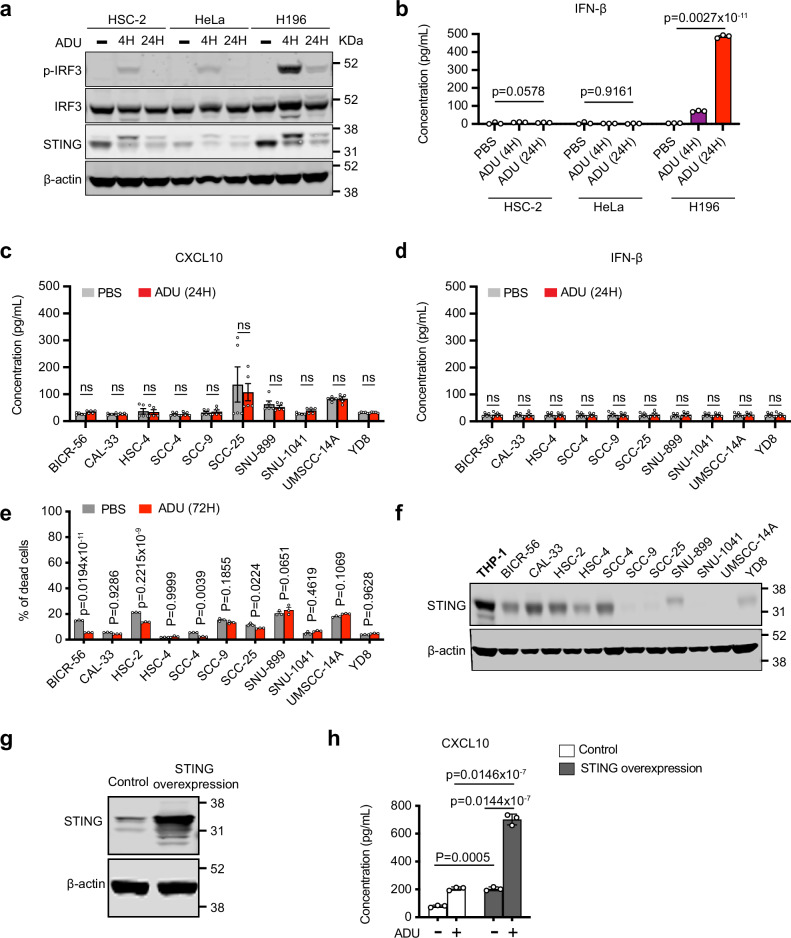
HNSCC cell lines exhibit reduced responsiveness to STING agonism. **a** Immunoblot of indicated proteins from HSC-2 cells, HeLa cells and H196 cells, with or without ADU (50 μM) treatment for 4 h (4H) and 24 h (24H). Data are representative of one independent experiment. **b** ELISA of IFN-β in conditioned media derived from **a** (three independent biological samples). ELISA of CXCL10 (**c**) and IFN-β (**d**) from multiple human HNSCC cell lines, with or without ADU (50 μM) treatment for 24H (five independent biological samples, pooled from two independent experiments). **e** Flow cytometric analysis of Annexin V and Helix NP of multiple human HNSCC cell lines, with or without ADU (50 μM) treatment for 72H. The percentage of dead cells indicates the proportion of Annexin V+ and/or Helix NP+ stained cells (three independent biological samples). **f** Immunoblot of STING in THP-1 and human HNSCC cell lines. Data are representative of one independent experiment. **g** Immunoblot of overexpression of STING in HSC-2 cells. Data are representative of one independent experiment. **h** ELISA of CXCL10 in conditioned media, with or without ADU (50 μM) treatment for 24 H in HSC-2 cells (three independent biological samples). Data in **b**, **c** and **h** were calculated by two-way ANOVA followed by Tukey’s multiple comparisons test. Data in **e** was calculated by two-way ANOVA followed by Šídák’s multiple comparisons test. Data are represented as mean ± SEM. ns, nonsignificant. Source data are provided as a Source Data file.

We first examined STING agonist response in the human HSC-2 HNSCC cell line, and compared results with a known non-responsive cancer cell line (HeLa cells, which undergo HPV18 E7–mediated STING silencing^27^) or a known responsive cancer cell line (H196 cells, a STING-positive non-neuroendocrine small cell lung cancer line^21,28^). We treated cell lines with high-dose ADU-S100 (ADU) (50 μM) for 4 or 24 h (H). HSC-2 cells and HeLa cells expressed lower levels of STING relative to H196 cells as expected (Fig. 1a). All three cell lines still exhibited clear STING turnover in response to ADU treatment, but HSC-2 and HeLa cells exhibited minimal p-IRF3 activation at 4H that was not sustained, in contrast to H196 cells (Fig. 1a). In consonance with these findings, ADU treatment of control H196 cells induced substantial secretion of IFN-β in conditioned media, whereas the same treatment of HSC-2 cells and HeLa cells did not (Fig. 1b). We also found that, in contrast to transfection with the dsDNA mimic poly(dG:dC), transfection with the dsRNA mimic Poly(I:C) substantially increased IFN-β production in HSC-2 cells after 24 h (Supplementary Fig. 1a). These findings are consistent with prior work suggesting that HNSCC cell lines may be relatively blunted in their response to STING agonism.

We therefore screened a larger panel of human HNSCC cell lines for ADU responsiveness. Treatment of BICR-56, CAL-33, HSC-4, SCC-4, SCC-9, SCC-25, SNU-899, SNU-1041, UMSCC-14A and YD8 cells with high-dose ADU also failed to induce secretion of CXCL10, a particularly sensitive marker of STING agonist activity, as well as IFN-β, across all cell lines examined (Fig. 1c, d). Since STING-IFN signaling can promote tumor cell cytotoxicity^14^, we also assessed the impact of high-dose ADU treatment on cell viability and apoptosis via Annexin V and Helix NP flow cytometry. As expected, we failed to observe induction of apoptosis in any of these human HNSCC cell lines (Fig. 1e and Supplementary Fig. 1b). Similar to HSC-2 cells, all these additional human HNSCC cell lines expressed relatively low levels of STING when compared with human monocytic cell line THP-1 cells that showed high sensitivity to ADU^20,24^ (Fig. 1f).

To further examine whether HNSCC cells might still be capable of responding to ADU in the presence of restored STING expression, we next overexpressed STING in HSC-2 cells and examined the response to the same high-dose ADU treatment. HSC-2 cells with enforced STING expression responded to ADU stimulation as measured by CXCL10 secretion, reinforcing the ability of ADU to cross the cancer cell membrane in HNSCC cells (Fig. 1g, h). Taken together, these data confirm that HNSCC cell lines generally express low levels of STING but are potentially able to respond to STING agonists when STING expression is elevated.

### STING agonist responses in HNSCC tumor explants are primarily immune cell driven

**Fig. 2:**
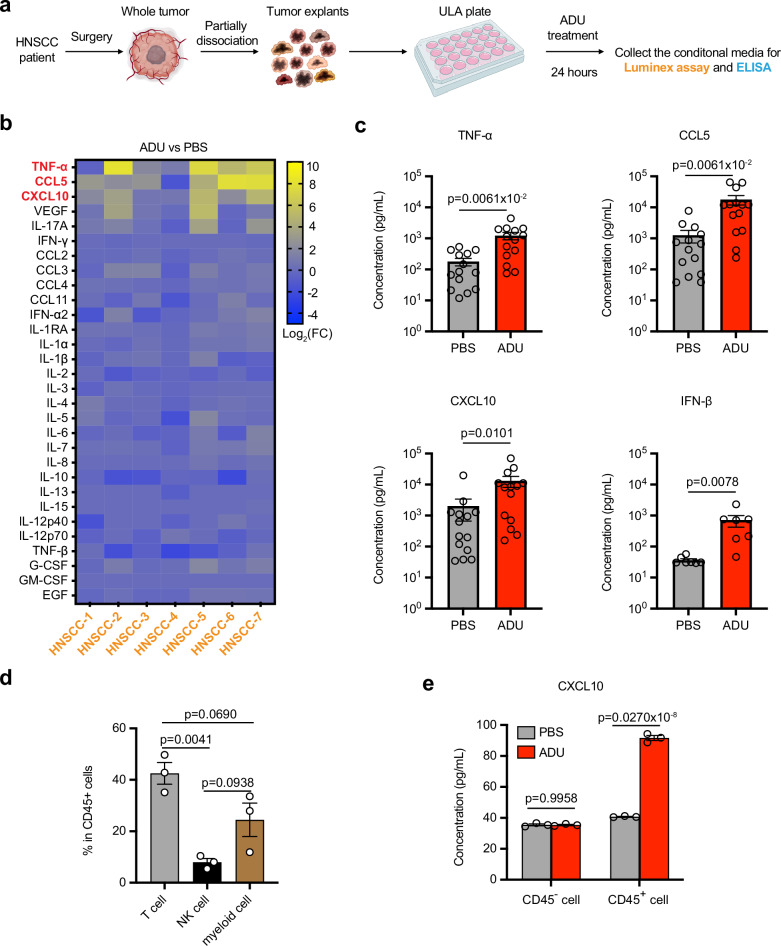
HNSCC tumor explant response to ADU is largely myeloid driven. **a** Schematic of protocol for HNSCC patient tumor explants (above 100 μm) dissociation and stimulation with ADU (50 μM) for 24 h in Ultra-Low Attachment (ULA) 24-well plate. Created in BioRender. Campisi, M. (2026) https://BioRender.com/nclt6gb. **b** Cytokine/chemokine heatmap analyzed by Luminex assay for conditioned media from tumor explants of HNSCC#1 to HNSCC#7. Value of Log_2_(FC of ADU vs PBS) was shown (two or three independent biological samples). FC, fold change. **c** Concentratio of TNF-α, CCL5 and CXCL10 analyzed by Luminex assay from **b** (HNSCC-1 to HNSCC-7), or ELISA (HNSCC-8 to HNSCC-14). Concentratio of IFN-β analyzed by ELISA (HNSCC-8 to HNSCC-14). Each dot represents one patient sample (*n* = 14 for TNF-α, CCL5 and CXCL10, *n* = 7 for IFN-β). **d** Flow cytometric analysis of tumor-infiltrating T cell (CD45^+^CD3^+^CD56^-^), NK cells (CD45^+^CD3^-^CD56^+^) and myeloid cells (CD45^+^HLA-DR^+^CD64^+^) in three distinct HNSCC patient tumors (HNSCC-15, HNSCC-16 and HNSCC-17). The percentage of each cell population was shown. **e** CD45^+^ and CD45^-^ cells were sorted from HNSCC-16 by flow cytometry and then treated with ADU (50 μM) for 24 h ELISA of CXCL10 was shown (three independent biological samples). Data in **c** was calculated by one-tailed Wilcoxon matched-pairs signed rank test, data in **d** was calculated by one-way ANOVA with Tukey’s multiple comparisons test, data in **e** was calculated by two-way ANOVA followed by the Šídák *t*-test. Data are represented as mean ± SEM. NS, nonsignificant. Source data are provided as a Source Data file.

We next sought to extend these findings beyond in vitro cell line models to human HNSCC tumor explants, which had previously been reported to respond to STING agonist CDN treatment ex vivo^4^. Portions of surgically resected HNSCC patient primary tumors were freshly isolated and partially dissociated into tumor explants (above 100 μm diameter) following our previously established protocol^29,30^ (Fig. 2a). We treated tumor explants from a cohort of seven patients (from HNSCC-1 to HNSCC-7) with control versus ADU for 24 h and collected conditioned media for multiplexed cytokine profiling (Fig. 2b). ADU treatment uniquely induced TNF-α, CCL5, and CXCL10 in HNSCC patient tumor explants (Fig. 2b). We confirmed these results in a second cohort of patient tumor explants (from HNSCC-8 to HNSCC-14) by testing TNF-α, CCL5, and CXCL10 via ELISA, observing their induction with ADU treatment (Fig. 2c). In addition, we found IFN-β was also significantly increased upon ADU treatment in HNSCC patient tumor explants, as expected (Fig. 2c).

To determine which specific cell populations in these explants produce these cytokines, we profiled their tumor microenvironment (TME) by flow cytometry and found that HNSCC explant models as expected contain a diversity of immune cell populations, including myeloid cells (CD45^+^HLA-DR^+^CD64^+^), T cell (CD45^+^CD3^+^CD56^-^), and NK cells (CD45^+^CD3^-^CD56^+^) besides tumor cells (CD45^-^EpCAM^+^EGFR^+^, not shown) (Fig. 2d).

We next sorted CD45^+^ and CD45^-^ populations, to broadly compare ADU response of immune cells to tumor and stromal cells. In consonance with our data in HNSCC cell line models, ADU treatment failed to induce CXCL10 from CD45^-^ cells, but did induce response from CD45^+^ immune cells (Fig. 2e). These data confirm that primary tumor HNSCC cells, similar to the HNSCC cell line models, are intrinsically refractory to STING agonism, which instead acts upon the immune TME.

### PTPN2 activity suppresses HNSCC tumor cell-intrinsic STING expression

Since restoring STING signaling in tumor cells is known to prime immunogenicity in other cancer types^14,22^, we next sought to dissect the mechanisms underlying suppression of STING in HNSCC tumor cells. We previously reported that *STING* transcription is suppressed in KRAS-LKB1 mutant lung cancer cells due to DNA hypermethylation and/or H3K27 tri-methylation, and that DNMT inhibition -/+ EZH2 inhibition can restore STING expression^14^. Additionally, TREX1 has also been identified as a suppressor of STING activation via STAT1-mediated control of STING mRNA transcription, limiting tumor cell STING-IFN signaling in multiple cancer types^18,31^. We therefore explored whether either of these mechanisms might be operative in HNSCC.

We first interrogated CpG methylation levels of *STING* promoter using data available from the Cancer Cell Line Encyclopedia (CCLE), and compared THP-1 cells (negative control) with the KRAS-LKB1 STING absent/promoter methylated lung cancer cell line A549 (positive control), and the HNSCC cell lines HSC-2, HSC-4, SCC-4, SCC-9, and SCC-25. As expected, we observed no hypermethylation of *STING* promoter regions in THP-1 cells, whereas A549 cells exhibited hypermethylation across the entire promoter. In contrast, HSC-2 and HSC-4 cells contained only one hypermethylated position, and none of the other cell lines displayed any DNA methylation of the *STING* promoter (Supplementary Fig. 2a). To validate these results functionally and assess STING promoter DNA histone H3K27 methylation, we next treated each cell line with DNMT inhibition (Decitabine) -/+ EZH2 inhibition (Tazemetostat) for 5 days. Consistent with previous results, this treatment restored STING expression in A549 cells^14^. By contrast, DNMT and EZH2 inhibition failed to increase STING in HNSCC cell lines, including HSC-2 and HSC-4 cells (Supplementary Fig. 2b). We next explored whether TREX1 might be repressing STING expression and thus generated TREX1 knockout HSC-2 cells by CRISPR-Cas9. Deletion of TREX1 also failed to restore STING activation in HSC-2 cells, thus diminishing the possibility that TREX1 is responsible for suppressing STAT1-mediated STING activation in HNSCC cells (Supplementary Fig. 2c,d).

**Fig. 3:**
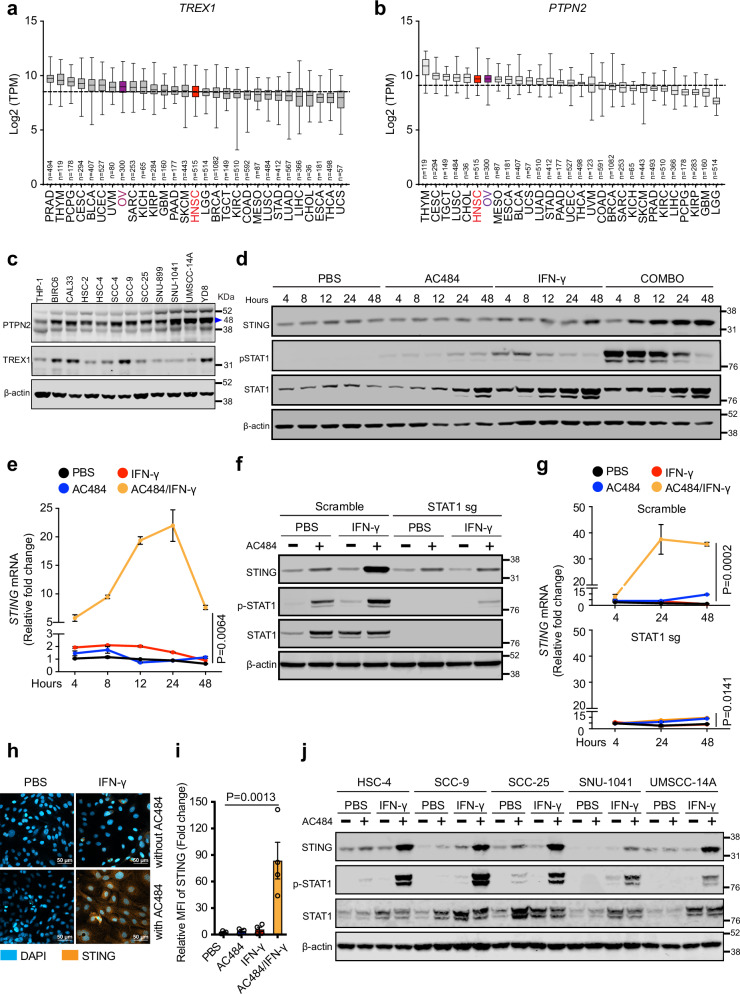
Combination AC484/IFNγ restores STING expression in HNSCCs. **a**, **b** Expression of PTPN2 and TREX1 among multiple malignancies from TCGA. OV, Ovarian serous cystadenocarcinoma. The abbreviations are based on TCGA study Abbreviations, https://gdc.cancer.gov/resources-tcga-users/tcga-code-tables/tcga-study-abbreviations. TPM, transcripts per million. The min-to-max box plot was shown. **c** Immunoblot of basal PTPN2 and TREX1 in THP-1 cells and multiple HNSCC cell lines. Data are representative of one independent experiment. **d** Immunoblot of indicated proteins in HSC-2 cells treated with IFN-γ (10 ng/mL) and AC484 (10 μM) for 4, 8, 12, 24 and 48 h. Data are representative of one independent experiment. **e** qPCR assay of STING in HSC-2 cells treated with IFN-γ (10 ng/mL) and AC484 (10 μM) for 4, 8, 12, 24, and 48 h (three independent biological samples). **f** Immunoblot of indicated proteins in HSC-2 cells transduced with the indicated vectors, treated with IFN-γ (10 ng/mL) and AC484 (10 μM) for 48 h. STAT1 sg (single guide RNA targeting STAT1). Two independent expreiments. **g** qPCR assay of STING in HSC-2 cells transduced with the indicated vectors, treated with IFN-γ (10 ng/mL) and AC484 (10 μM) for 4, 24 and 48 h (three independent biological samples). **h** Confocal microscopy imaging of STING in HSC-2 cells from indicated groups, treated with IFN-γ (10 ng/mL) and AC484 (10 μM) for 48 h (four independent biological samples). **i** Quantification of STING level (normalized to DAPI) in **h** by ImageJ software (*n* = 3 or 4 independent fields. *n* = 3 for AC484 group, *n* = 4 for PBS, IFN-γ and AC484/IFN-γ groups). MFI, mean fluorescence intensity. **j** Immunoblot of indicated proteins from HSC-4, SCC-9, SCC-25, SNU-1041, and UMSCC-14A cells, treated with IFN-γ (10 ng/mL) and AC484 (10 μM) for 48 h. Data are representative of one independent experiment. Data in **e** and **g** were calculated by two-way ANOVA followed by Tukey’s multiple comparisons test; data in **i** was calculated by one-way ANOVA followed by Tukey’s multiple comparisons test. Data are represented as mean ± SEM. Source data are provided as a Source Data file.

To explore whether other unique mechanisms could be suppressing STING expression/activity in HNSCC, we next considered whether other negative regulators of STAT1 signaling might be activated. Interestingly, while analysis of *TREX1* mRNA levels in The Cancer Genome Atlas (TCGA) revealed moderate expression in HNSCC on average compared with other cancer types, HNSCC and ovarian (OV) cancer mRNA levels of PTPN2 were among the highest of all solid tumors (Fig. 3a, b). To confirm this finding in HNSCC cell lines, we compared PTPN2 and TREX1 protein levels, alongside THP-1 cells as control. While THP-1 cells expressed modest levels of both PTPN2 and TREX1, PTPN2 was broadly increased across HNSCC cell lines, in contrast to more variable TREX1 expression (Fig. 3c). Since PTPN2 acts to repress STAT1 activation, these data suggest that PTPN2 upregulation could potentially represent a mechanism of tumor cell STING suppression.

To explore whether PTPN2 might be responsible for repressing STING expression in HNSCC, we next treated cells with the PTPN2/1 inhibitor AC484. Since PTPN2 restrains IFN-γ -STAT1 signal transduction, we also included treatment conditions with IFN-γ alone, or in combination with AC484 treatment. Interestingly, whereas STING levels were not substantially induced by IFN-γ or AC484 treatment alone, the combination resulted in robust increase in STING protein levels over time, which correlated with induction of p-STAT1 (Fig. 3d). We also noted that despite PTPN2/1 inhibition, additional negative feedback mechanisms were still able to revert p-STAT1 activation at 48 h, the time point at which STING protein levels peaked. Furthermore, STING mRNA levels were also potently stimulated by the combination of IFN-γ with AC484, peaking at 24 h and then falling at 48 h (Fig. 3e). This result was also specific to IFNγ treatment, since IFN-β and AC484 combination treatment minimally induced p-STAT1 and STING expression as compared with IFN-γ plus AC484, and the combination of IFN-β and IFN-γ plus AC484 did not further augment STING levels (Supplementary Fig. 3a).

To assess whether STAT1 activation is directly responsible for the induction of STING expression following IFN-γ and AC484 treatment, we next generated STAT1-deficient HSC-2 cells by CRISPR-Cas9 and compared the results with cells expressing a control guide RNA. We again confirmed a pronounced increase in STING protein expression mediated by the combination of IFN-γ and AC484 treatment in control cells, which was completely abrogated by STAT1 knockout (Fig. 3f). To understand if this is controlled primarily at the level of *STING* gene transcription, we also examined STING mRNA induction following treatment with the combination, and observed robust suppression of this phenotype in STAT1-deficient HSC-2 cells (Fig. 3g). To dissect whether PTPN2 or PTPN1 mediates this effect, since AC484 targets both proteins, we generated single or double knockout HSC-2 cells via CRISPR-Cas9 (Supplementary Fig. 3b–d), confirming that the major PTPN2 isoform in HSC2 cells is 48 kDa, equivalent to A549 cells known to express this same dominant isoform^32,33^ (Supplementary Fig. 3b, c). We further determined that specific deletion of this PTPN2 isoform, but not PTPN1, plays the major role in STING induction and p-STAT1 activation (Supplementary Fig. 3e), and that PTPN2 genetic deficiency was equivalent to the effects of AC484 treatment in promoting IFN-γ–induced STING expression at both the protein and mRNA level (Supplementary Fig. 3f, g). Together, these data confirm that PTPN2-mediated inhibition of STAT1 activation primarily represses STING expression transcriptionally, by suppressing its mRNA induction.

We next considered whether this effect might occur in a subpopulation of cancer cells uniquely or whether it is generalizable across the entire population of HSC-2 cells. IFN-γ and AC484 combination treatment induced STING homogeneously across HSC-2 cells, as measured by indirect immunofluorescence, in contrast to IFN-γ or AC484 alone which only occasionally induced STING expression in individual cells (Fig. 3h, i). We also examined the impact of IFN-γ and AC484 combination treatment across multiple additional human HNSCC cell lines, including those with particularly low STING levels such as HSC-4, SCC-9, SCC-25, SUN-1041, and UMSCC-14A (Fig. 1h). p-STAT1 and STING levels were consistently induced by combination treatment across HNSCC cell lines examined, further confirming the generalizability of this result (Fig. 3j). Notably, SNU-1041 showed an increase in STING, but the total levels remained modest. This effect was also conserved across species, as IFN-γ and AC484 combination treatment increased STING levels in multiple syngeneic mouse HNSCC cell lines (Supplementary Fig. 3h–j). Finally, we also examined whether AC484 plus IFN-γ could promote STING induction in ovarian cancer, which also exhibited high PTPN2 expression (Fig. 3a). Indeed, we also observed STING de-repression by this combination in human ovarian cancer cell lines SKOV3 and COV318, and in another patient-derived ovarian cancer cell line, DF68^34^ (Supplementary Fig. 3k–m). Together, these data support a unique mechanism active in HNSCC and likely other cancer types whereby elevated PTPN2 activity restrains STAT1-mediated induction of STING expression.

### AC484/IFNγ-mediated induction of tumor cell STING unleashes ADU sensitivity

**Fig. 4:**
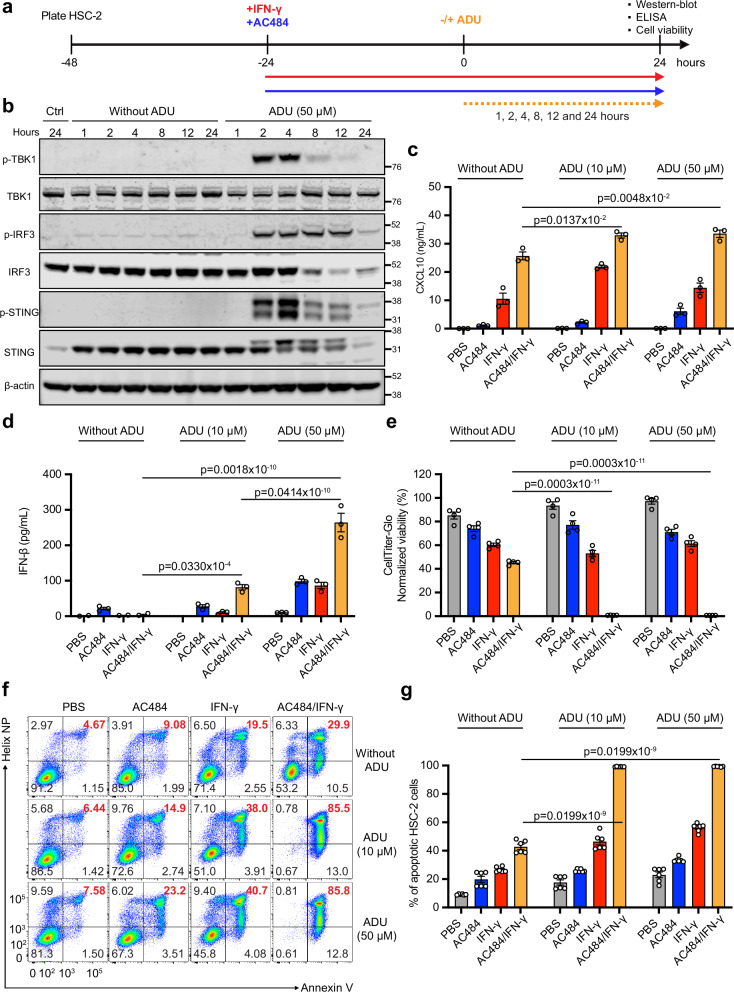
AC484/IFNγ-mediated STING induction restores ADU sensitivity. **a** Schematic of sequential drug treatment. HSC-2 cells were pretreated with IFN-γ (10 ng/mL) and AC484 (10 μM) for 24 h, and ADU (50 μM) for additional 1, 2, 4, 8,12 and 24 h. **b** Immunoblot of indicated proteins from HSC-2 cells treated with drugs as shown in **a**, HSC-2 cells without drug treatment as control (Ctrl). Data are representative of one independent experiment. **c** ELISA for CXCL10 derived from conditioned media from HSC-2 cells that were pretreated with IFN-γ (10 ng/mL) and AC484 (10 μM) for 24 h, and then ADU (10 μM and 50 μM) for additional 24 h (three independent biological samples). **d** ELISA for IFN-β derived from conditioned media of in **c** (three independent biological samples). **e** CellTiter-Glo assay for HSC-2 cell viability treated as in **c** (four independent biological samples). **f** Representative flow cytometry plots were shown. HSC-2 cells were treated as in **c** and stained with Annexin V and Helix NP. The percentage of apoptotic cells indicates the proportion of Annexin V+ and/or Helix NP+ stained cells. **g** Quantification of the percentage of apoptotic HSC-2 cells in **f** (six independent biological samples, combined from two independent experiments). Two independent experiments (**d**–**g**). Data in **c**–**e** and **g** were calculated by two-way ANOVA followed by Tukey’s multiple comparisons test. Data are represented as mean ± SEM. Source data are provided as a Source Data file.

We next considered whether unleashing STING expression downstream of IFN-γ and PTPN2i resensitizes HNSCC cells to STING agonist treatment. We therefore pre-treated HSC-2 cells with AC484 and IFN-γ for 24 h to restore STING expression, and then stimulated HSC-2 cells -/+ ADU for different periods of time (Fig. 4a). Without concurrent ADU treatment, the AC484 and IFN-γ increased STING protein level did not promote STING activation on its own, as measured by p-STING, p-TBK1 and p-IRF3 induction (Fig. 4b). In contrast, ADU treatment following AC484/IFN-γ-mediated STING induction markedly increased p-IRF3 within 2 to 12 h as well as STING phosphorylation and eventual turnover at 24 h (Fig. 4b). Together these data indicate that unleashing STING expression by reactivating STAT1 signaling restores ADU sensitivity in HNSCC cells.

Previously, it was reported that PTPN2 could promote STING proteasomal degradation by mediating its dephosphorylation^35^. Thus, in addition to regulating STING transcriptionally, AC484 could also prevent STING turnover. To address the possibility, we performed the same experiment in which HSC-2 cells were pre-treated with AC484 and IFN-γ for 24 h to increase STING levels, but following washout, we replenished cells with either ADU+IFN-γ alone or with ADU+AC484/IFN-γ (Supplementary Fig. 4a). We observed equivalent STING turnover following ADU treatment in the absence or presence of continued PTPN2i (Supplementary Fig. 4b). Thus, PTPN2 does not appear to regulate HNSCC tumor cell STING turnover, at least in this context.

We next evaluated the consequences of restored tumor cell STING expression and activation on HSC-2 cell viability, as well as induction of CXCL10 and type I IFN. In the absence of ADU stimulation, AC484/IFN-γ treatment failed to increase IFN-β levels, consistent with the lack of p-IRF3 induction (Fig. 4c, d). In contrast, ADU treatment strongly promoted CXCL10 and IFN-β production from AC484/IFN-γ treated HSC-2 cells, using both low and high ADU concentrations (10 μM vs 50 μM) (Fig. 4c, d). Similarly to ADU, poly(dG:dC), the repetitive synthetic double-stranded DNA, also increased IFN-β production from AC484/IFN-γ–treated HSC-2 cells (Supplementary Fig. 5a). We also confirmed that AC484/IFN-γ pretreatment restored ADU-induced IFN-β and CXCL10 from mouse HNSCC cell lines and human ovarian cancer SKOV3 cells and DF68 (Supplementary Fig. 5b–g). Notably, AC484/IFN-γ priming of HSC-2 cells strongly enhanced ADU-induced cytotoxicity (Fig. 4e), such that even reduced concentrations of ADU (10 μM) were able to potentiate massive HSC-2 cell apoptosis equivalent to high-dose ADU (50 μM) (Fig. 4f, g). Thus, unleashing tumor cell STING expression in HNSCC cells restores ADU-induced STING activation and the production of CXCL10 and IFN-β, resulting in substantial cellular toxicity.

### NK cell-derived IFN-γ potentiates AC484-mediated tumor cell STING activation

**Fig. 5:**
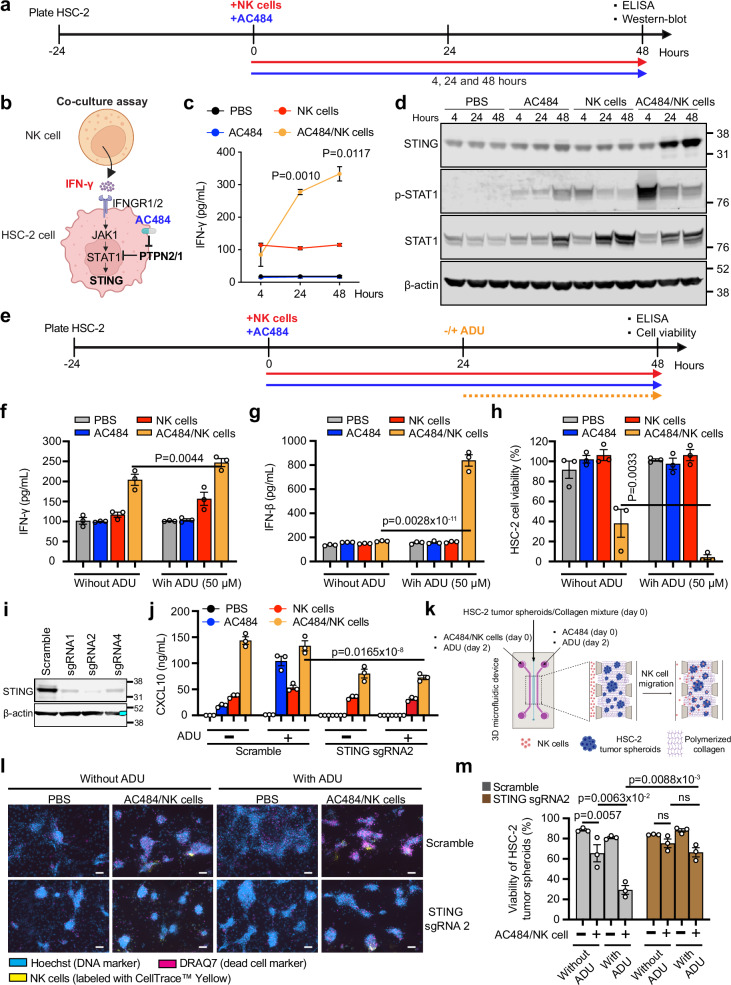
NK cell derived IFN-γ cooperates with AC484 to restore tumor cell STING signaling. **a** Schematic of drug treatment. PBMC-derived NK cells (10 K) and HSC-2 cells (100 K) were co-cultured at 1:10, with or without AC484 (10 μM) for 4, 24 and 48 h. **b** Schematic of NK cells and HSC-2 cells co-culture assay. Created in BioRender. Campisi, M. (2026) https://BioRender.com/lkw0fat. **c** ELISA of IFN-γ from the conditioned media derived from the co-culture assay in **a** (three independent biological samples). **d** Immunoblot of indicated proteins in HSC-2 cells treated as in **a**. **e** Schematic of sequential drug treatment. HSC-2 cells (100 K) were pretreated with PBMC-derived NK cells (10 K) and AC484 (10 μM) for 24 h, and then with or without ADU (50 μM) for additional 24 h. **f** ELISA of IFN-γ from the conditioned media derived from **e** (three independent biological samples). **g** ELISA of IFN-β from the conditioned media derived from **e** (three independent biological samples). **h** CTG assay for HSC-2 cells treated as **e** (three independent biological samples). **i** Immunoblot of STING proteins in HSC-2 cells transduced with the indicated vectors. Data are representative of one independent experiment. Data are representative of one independent experiment. **j** ELISA for CXCL10 derived from conditioned media from indicated HSC-2 cells that were pretreated with IFN-γ (10 ng/mL) and AC484 (10 μM) for 24 h, and then ADU (50 μM) for additional 24 h (three independent biological samples). **k** Schematic of NK cell migration and killing assay in 3D microfluidic device. Created in BioRender. Campisi, M. (2026) https://BioRender.com/hjvu7c5. **l** Representative immunofluorescence images of live/dead staining for HSC-2 tumor spheroids from indicated groups. Scale bar: 100 μm (three independent biological samples). **m** Viability of HSC-2 tumor spheroids (80 × 10^3^) sequentially treated with AC484 (10 μM)/NK cells (20 × 10^3^) and ADU (50 μM) as shown in **k** (three independent biological samples). Data represent two independent experiments (**f**–**h**). Data in **c**, **f**, **g**, **j**, **h** and **m** were calculated by two-way ANOVA followed by Tukey’s multiple comparisons test. Data are represented as mean ± SEM. Source data are provided as a Source Data file.

Both AC484 and ADU treatments efficiently promote NK cell infiltration into the TME in syngeneic mouse tumor models^26,36^, and NK cells are resistant to ADU-induced toxicity^8^. We therefore next explored whether NK cell-derived IFN-γ plus AC484 could increase STING in HNSCC tumor cells, eliminating the need to supplement IFN-γ directly in the culture media. We co-cultured HSC-2 cells and NK cells isolated from human peripheral blood mononuclear cells (PBMCs), with or without AC484 for different time courses (Fig. 5a, b). We observed that IFN-γ concentrations gradually increased only in the AC484/NK cell combination group from 4 to 48 h (Fig. 5c). Also, NK cells alone treated with AC484 for 48 h showed enhanced IFN-γ production (Supplementary Fig. 6a). Furthermore, in consonance with our findings above, we observed upregulation of STING in HSC-2 cells specifically upon generation of NK cell-derived IFN-γ following AC484 treatment (Fig. 5d). Thus, NK cells co-cultured with HNSCC cells are able to produce IFN-γ upon PTPN2i, restoring tumor cell STING expression to an equivalent degree as exogenously added IFN-γ.

To examine whether the STING induction by NK cell-derived IFN-γ and AC484 might also sensitize tumor cells to STING agonism, HSC-2 cells were pre-treated with NK cells and AC484 for 24 h to increase STING, followed by ADU or control treatment for an additional 24 h (Fig. 5e). Addition of ADU further potentiated IFN-γ induction by NK cells combined with AC484 (Fig. 5f) and uniquely resulted in the generation of IFN-β in the setting of this combination (Fig. 5g). Furthermore, consistent with the induction of IFN-γ, NK cells combined with AC484 induced more than 50% HSC-2 cell apoptosis in the AC484/NK cells group (Fig. 5h and Supplementary Fig. 6b–d). Of note, AC484 was not toxic to NK cells (Supplementary Fig. 6b). Further addition of ADU following NK cell and AC484 pre-treatment again resulted in profound HSC-2 cell death (Fig. 5h), matching our results with exogenous IFN-γ (Fig. 4f, g). We also observed similar results from the SKOV3 cell model (Supplementary Fig. 6e, f). Thus, NK cells can provide a physiologic source of IFN-γ that potentiates AC484-mediated upregulation of STING in HNSCC cells, resensitizing them to STING agonism.

To further validate the role of tumor cell intrinsic STING in tumor immunogenicity, we generated STING knockout HSC-2 cells by CRISPR-Cas9 (Fig. 5i). As expected, STING knockout impaired CXCL10 production upon AC484/IFN-γ/ADU treatment as compared with control cells (Fig. 5j). We next used a 3D microphysiologic cell culture model to directly assess the contribution of tumor cell STING to AC484- and ADU-mediated NK cell activity in killing HSC-2 tumor spheroids^8,18,29,30^. NK cells were added to the left side channel of the microfluidic device on day 0, adjacent to HSC-2 tumor spheroids embedded in collagen, -/+ AC484 and ADU treatment sequentially on day 0 and day 2, respectively (Fig. 5k). In consonance with our findings in 2D culture, AC484 treatment alone slightly enhanced NK cell killing of control HSC-2 tumor spheroids, while the combination of AC484 followed by ADU significantly enhanced HSC-2 cell death (Fig. 5l, m). In contrast, STING-deficient HSC-2 tumor spheroids were resistant to cell death upon exposure to NK cells in the presence of AC484/ADU (Fig. 5l, m). Together, these findings reveal that restoration of tumor cell STING signaling is causally linked to the enhanced NK cell killing mediated by AC484 and ADU treatment.

### Combined treatment with STING agonism and AC484 suppresses HNSCC tumor growth in vivo

**Fig. 6:**
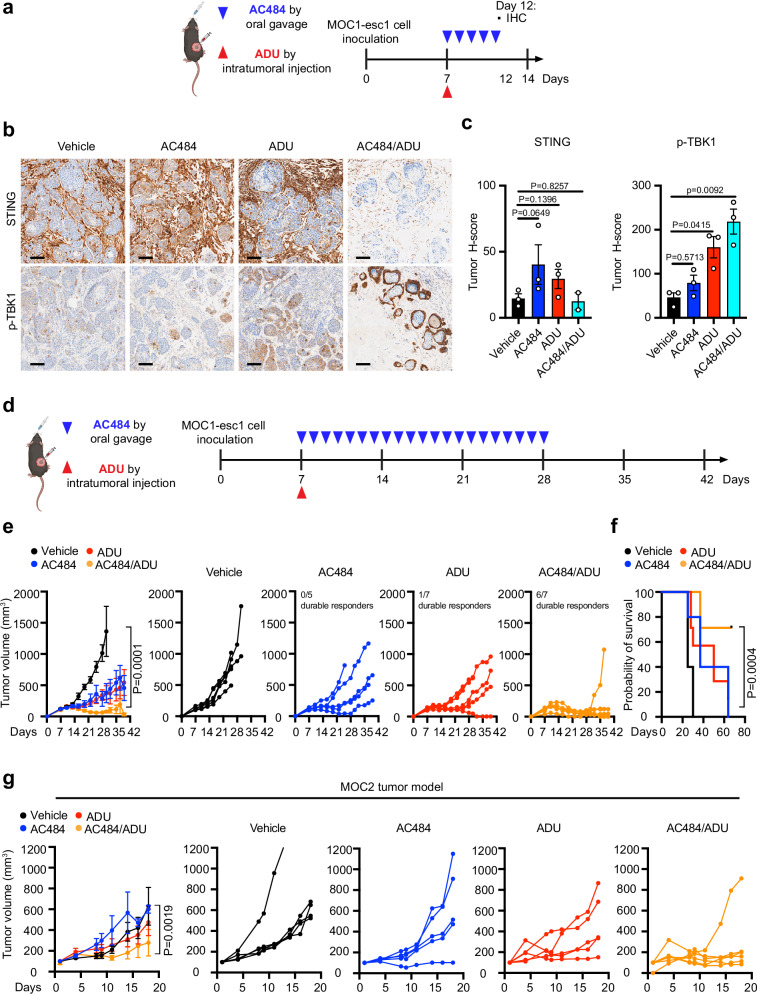
Combined treatment with STING agonism and AC484 suppresses HNSCC tumor growth in vivo. **a** Schematic of pharmacodynamics study with ADU (20 μg) and AC484 (50 mg/kg) in syngeneic MOC1-esc1 model. Created in BioRender. Campisi, M. (2026) https://BioRender.com/49luw72. **b** Representative IHC images from tumor cell STING and p-TBK1 from mice treated as in **a** on day 12. Scale bar: 100 μm. Data are representative of one independent experiment. **c** Quantification of tumor cell STING and p-TBK1 in **b** by H-score (*n* = 3 mice for each group). **d** Schematic of pharmacodynamics study with ADU (20 μg) and AC484 (50 mg/kg) in syngeneic MOC1-esc1 model. Created in BioRender. Campisi, M. (2026) https://BioRender.com/bsc0l73. **e** Tumor volume of MOC1-esc1 tumor from mice treated as in (**d**) (*n* = 5 mice for Vehicle and AC484 group; *n* = 7 mice for ADU and AC484/ADU combination group). **f** Probability of survival of mice from **e**. **g** Tumor volume of MOC2 tumor from mice treated as in **d** (*n* = 5 mice per group). Data in **c** was calculated by one-way ANOVA followed by uncorrected Dunn’s multiple comparisons test, data in **e** and **g** were calculated by two-way ANOVA followed by Tukey’s multiple comparisons test, data in **f** was calculated by Log-rank (Mantel-Cox) test. Data are represented as mean ± SEM. Source data are provided as a Source Data file.

Next, we utilized the well-established MOC1-esc1 syngeneic anti-PD-1 resistant HNSCC mouse model to explore how PTPN2i might potentiate STING agonist response in vivo^37–39^. MOC1-esc1 tumors are highly infiltrated by Tregs and M2-like tumor-associated macrophages^39^. MOC1-esc1 tumors were established subcutaneously in C57BL/6 mice, and then AC484 (50 mg/kg) was given by oral gavage daily for 3 weeks, with or without a single dose of ADU (20 μg) administered by intratumoral injection (Fig. 6a). To examine tumor cell intrinsic STING activation in vivo, we collected tumor tissues on day 12 and tested tumor cell STING and p-TBK1 by immunohistochemistry (IHC). AC484 monotherapy moderately increased tumor cell STING, suggesting that alteration of the immune TME was sufficient to provide IFN-γ stimulation in vivo (Fig. 6b, c). As expected, AC484/ADU combination therapy exhibited decreased STING levels, consistent with ADU-induced STING turnover, while showing potent downstream pathway activation as measured by strong p-TBK1 induction (Fig. 6b, c).

Although AC484 or ADU monotherapy treatment partially suppressed tumor growth compared with the vehicle group, none of the mice from the AC484 group (0/5) and only one mouse from the ADU group (1/7) showed durable tumor control. In contrast, combination therapy led to potent and durable regression of tumor growth in the majority of mice (6/7) as well as increased probability of survival (Fig. 6d–f). Consistently, AC484/ADU combination therapy also suppressed tumor growth in another anti-PD-1 resistant HNSCC MOC2 tumor model, where 1/5 mice from the AC484 and ADU monotherapy group exhibited durable tumor control, but 4/5 mice from the AC484/ADU combination therapy showed persistent tumor control (Fig. 6g).

### AC484 potentiates STING agonist response in an immune cell-dependent manner

**Fig. 7:**
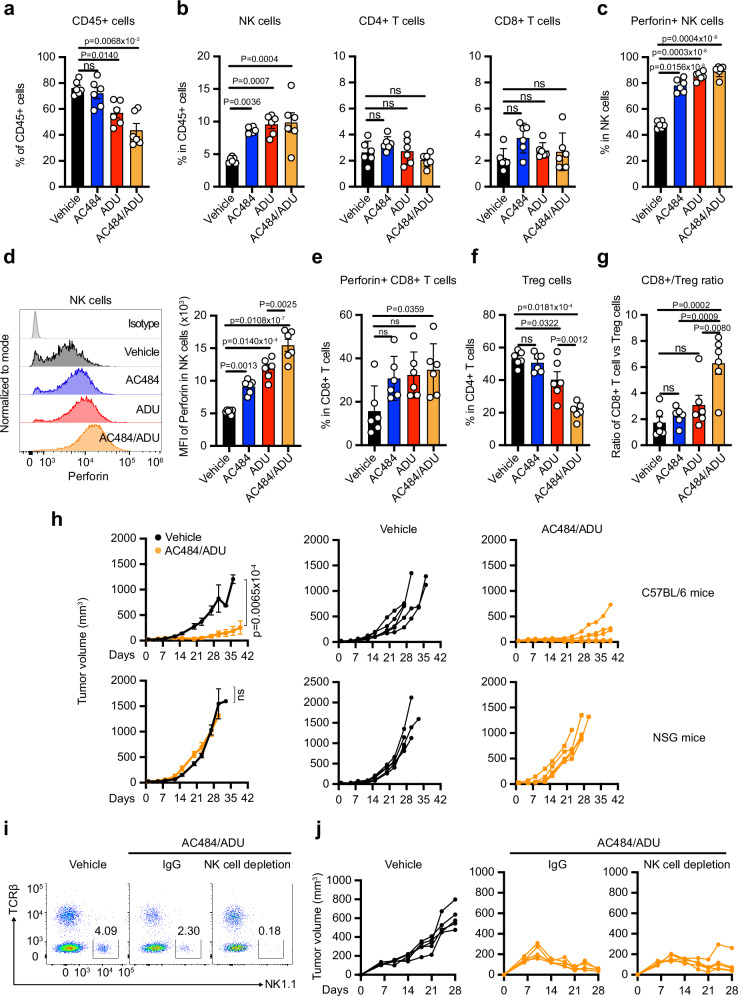
Potentiation of STING agonist response by AC484 is immune cell dependent. **a** Flow cytometric analysis of infiltrating immune cells in MOC1-esc1 tumors isolated on day 12, treated as in Fig. 6a. Quantification of viable CD45^+^ cells was shown (*n* = 6 mice for each group). **b** The percentage of NK cells (TRCβ^-^NK1.1^+^), CD4^+^T cells (TRCβ^+^NK1.1^-^CD4^+^CD8^-^), and CD8^+^T cells (TRCβ^+^NK1.1^-^CD4^-^CD8^+^) in viable CD45^+^ cells from **a** was shown (*n* = 6 mice for each group). **c** The percentage of perforin^+^ NK cells was shown (*n* = 6 mice for each group). **d** The MFI of perforin in NK cells was shown (Left) and quantified (Right) (*n* = 6 mice for each group). **e** The percentage of perforin^+^ CD8^+^T cells was shown (*n* = 6 mice for each group). **f** The percentage of Treg cells (Foxp3^+^CD4^+^) in CD4^+^T cells was shown (*n* = 6 mice for each group). **g** The ratio of CD8^+^T cells vs Treg cells was shown (*n* = 6 mice for each group). **h** Tumor volume of MOC1-esc1 tumor from C57BL/6 and NSG mice treated as in Fig. 6d (*n* = 5 mice for each group). **i** NK cell (CD45 + TRCβ^-^NK1.1^+^) depletion efficiency in peripheral blood of mice bearing with MOC1-esc1 tumor (*n* = 5 mice for each group). **j** Tumor volume of MOC1-esc1 tumor from mice from indicated groups in **i**. Data in **a**–**g** were calculated by one-way ANOVA followed by Tukey’s multiple comparisons test, data in **h** were calculated by two-way ANOVA followed by Tukey’s multiple comparisons test. Data are represented as mean ± SEM. Source data are provided as a Source Data file.

To examine which immune cell types are required for AC484/ADU-mediated tumor response, we profiled tumor-infiltrating immune cells in MOC1-esc1 tumors by flow cytometry on day 12, which revealed that overall CD45+ immune cell abundance was decreased in the ADU monotherapy and AC484/ADU combination therapy group (Fig. 7a). AC484 and ADU treatment each increased NK cell infiltration (Fig. 7b and Supplementary Fig. 7a) as expected^26,36^. The percentage of macrophages was slightly decreased in the combination therapy, while the percentage of CD4+ T cells, CD8+ T cells and dendritic cells was not changed (Fig. 7b and Supplementary Fig. 7a–c). We further examined NK cell and CD8+ T cell activation status by assessing perforin-positive cells. AC484 and ADU treatment alone, and the AC484/ADU combination group, showed increased percentage of perforin+ NK cells compared to control (Fig. 7c and Supplementary Fig. 7a). In addition, the mean fluorescence intensity (MFI) of perforin in NK cells was increased with AC484 and ADU combination therapy (Fig. 7d). Similarly, the percentage of perforin+ in CD8+ T cells was also increased after AC484 and ADU treatment (Fig. 7e). Interestingly, the percentage of Foxp3 + CD4+ regulatory T (Treg) cells was significantly decreased in ADU group (Fig. 7f and Supplementary Fig. 7a) and the percentage of Treg cells was further decreased in the AC484/ADU group compared with the ADU group (Fig. 7f), indicating that AC484 enhanced ADU-induced Treg cell loss. Thus, the ratio of CD8+ T cells vs Treg cells was significantly increased in the AC484/ADU group (Fig. 7g).

We next compared the impact of combined AC484/ADU treatment on MOC1-esc1 tumor growth in syngeneic mice as compared with NSG mice lacking mature T cells, B cells, and NK cells. In contrast to C57BL/6 mice, AC484/ADU treatment did not eradicate MOC1-esc1 tumor in NSG mice, indicating the importance of immune cells generally in AC484/ADU-mediated tumor suppression (Fig. 7h). To further analyze the unique contribution of NK cells in controlling MOC1-esc1 tumor growth, we also depleted NK cells via use of a blocking antibody in C57BL/6 mouse hosts (Fig. 7i). Despite successful depletion of NK cells (Fig. 7i), we observed that the loss of NK cell alone did not reverse tumor control in the AC484/ADU-treated group (Fig. 7j). These data indicate that the Treg depletion and/or other additional immunologic factors induced by AC484/ADU in vivo beyond NK cells also contributes to anti-tumor activity.

Together, these data confirm that AC484 treatment induces tumor cell STING expression in vivo, and, when combined with therapeutic STING agonism, results in pronounced immune dependent control of HNSCC tumor growth (Supplementary Fig. 8). These data thus further validate the ability of PTPN2i to restore HNSCC immunogenicity and unleash tumor cell STING expression in vivo, while also demonstrating the therapeutic rationale for combining AC484 with STING agonism in the clinic.

## Discussion

Beyond the well-established role of epigenetic silencing of the STING locus, we and others have shown that the strength of IFN-STAT1 signaling strength also regulates tumor cell STING induction and activation^40^. In parallel, PTPN2 has been shown to prevent IFN-STAT1 signaling, and a potent PTPN2/PTPN1 active-site inhibitor, AC484, unleashes STAT activity and promotes immune-mediated antitumor activity^25,26^. With this background, we discover here that the combination of AC484 with IFN-γ uniquely and efficiently increases HNSCC tumor cell STING expression. IFN-γ treatment alone increases p-STAT1 over the short-term (within hours) but is limited by negative feedback^41^. Indeed, in consonance with PTPN2 representing a dominant negative regulator of STAT1 activation in HNSCC cells, we observed increased PTPN2 expression across HNSCC tumors and cell lines, as well as robust prolongation of p-STAT1 induction beyond 48 h when AC484 treatment was combined with IFN-γ. Furthermore, the induction of STING expression by this combination was clearly STAT1 dependent, since STAT1-deficient HSC-2 cells showed defective STING induction upon AC484/IFN-γ treatment (Fig. 3f, g). These findings further corroborate the role of STAT1 signaling in regulating tumor cell STING induction and identify PTPN2 upregulation as a mechanism capable of repressing STING expression.

Pharmacologic activation of the cGAS-STING pathway by STING agonists has not been successful to date in clinical trials^6,7,42^. A major reason may be that current STING agonists may activate STING signaling in myeloid cells bearing high STING in the TME, but not affect cancer cells expressing low STING^12^. High dose STING agonism also causes T cell toxicity, thereby limiting the activity of the key immune effector cell population required for tumor eradication^8–11^. Restoring tumor cell sensitivity to STING agonists by increasing tumor cell intrinsic STING levels thus provides a promising biomarker-driven method to resensitize cancer cells and may overcome the current limitations in targeting this pathway. Beyond enhancing antigen presentation, cell intrinsic STING activation can induce cell death, as well as the production of type I interferons, chemokines, and other cytokines that prime the TME and recruit effector T and NK cells. Indeed, we confirmed that AC484/IFN-γ treatment restored IFN-β production in ADU concentration-dependent manner (Fig. 4d). Moreover, even low concentrations of ADU (10μM) efficiently induced HSC-2 cell apoptosis (Fig. 4e–g) below those that efficiently induce T cell death^8–11^. Thus, priming STING activation in HNSCC tumor cells with AC484/IFN-γ amplifies sensitivity of cancer cells to even low-dose STING agonism, not only expanding its activity in the TME but also potentially limiting T cell toxicity.

Indeed, in vivo experiments revealed that combination treatment of ADU with AC484 was more effective at eradicating anti-PD-1-resistant HNSCC mouse tumors (MOC1-esc1 and MOC2), with recruitment of NK cells sufficient to provide a source of IFN-γ in the TME. We also found that ADU/AC484 combination therapy significantly decreased the percentage of Treg cells (Fig. 7f). Type I interferon signaling is known to attenuate regulatory T-cell function^43^, and thus ADU/AC484 combination therapy could indirectly induce Treg cell death indirectly by inducing type I interferon production in the TME. Furthermore, although ADU/AC484 combination treatment enhanced perforin production in both NK and CD8+ T cells (Fig. 7c–e), and enhanced infiltration of NK cells into tumors, we did not observe increased numbers of CD8+ T cells (Fig. 7b). However, we selected an early time point (day 12) following ADU treatment, whereas expansion of clonal CD8+ T cells that ultimately mediate durable tumor rejection likely occurs on a longer time scale.

Our study has several important limitations. First, although we clearly demonstrate a role for NK cells in promoting AC484/ADU activity in vitro and in 3D models, and observed increased NK cell infiltration and activation in vivo, depletion of NK cells alone was not able to reverse activity, in contrast to the effects observed with combination treatment in NSG mice. These data suggest multifactorial involvement of immune cells in vivo mediating AC484/ADU activity, potentially involving the Treg depletion highlighted above. Second, because it is difficult to measure cell-type-specific IFN-γ production in vivo, it is also challenging to isolate other potential sources of this cytokine beyond NK cells in the TME. Future work using single-cell RNA sequencing at different timepoints in vivo will help to define the full breadth of immune cell populations activated by AC484/ADU combination therapy that contribute to anti-tumor responsiveness. Finally, ADU-S100 has important pharmacologic limitations as a first-generation STING agonist. Thus, next-generation systemic STING agonists, including novel formulations as payloads on tumor cell targeting antibody drug conjugates^44^, have substantially greater therapeutic potential to combine with PTPN2/1 inhibitors in the clinic.

ICB has revolutionized the standard of care therapies for several tumors, including for advanced HNSCC. However, the response rate with standard of care of PD-1 ICB monotherapy in patients with advanced HNSCC is less than 20%^45^. Novel immunotherapeutic approaches to increase the efficacy of cancer immunotherapy are clearly still needed. We previously reported that a combination of EZH2 inhibitors with PD-1 blockade efficiently suppresses MOC1-esc1 tumor growth by enhancing MHC class I upregulation and promoting antigen-specific CD8+ T cell function^38^. Here, we show that combination therapy of PTPN2/1 inhibitor, AC484 and STING agonist, ADU, significantly impairs MOC1-esc1 and MOC2 tumor growth by inducing tumor cell-intrinsic STING induction and activation, providing an alternative strategy for overcoming anti-PD-1 resistance in HNSCC. Currently, the safety and efficacy of AC484 are being evaluated in a phase 1 trial enrolling patients with locally advanced or metastatic tumors, including HNSCC (ClinicalTrials.gov identifier NCT04777994). Based upon our findings, combination therapy with AC484 and STING agonism has the future potential to augment activity and overcome anti-PD-1 resistance in HNSCC and possibly additional cancer types.

## Methods

### Cell lines

Human HNSCC cell lines BICR-56, CAL33, HSC-2, HSC-4, SCC-4, SCC-9, SCC-25, SNU-899, SNU-1041, UMSAA-14A, and YD8 cell lines were obtained from Peter Hammerman lab (DFCI). HSC-2, SCC-25, SNU-899, YD8 and HeLa were authenticated by Short Tandem Repeats (STRs) genotyping. Lung cancer cell line A549 was obtained from the Broad Institute and authenticated by STRs. All these cell lines were cultured in Dulbecco’s Modified Eagle Medium (DMEM) (Corning, # 10-013-CV) containing 10% fetal bovine serum (FBS) (Gemini Bio-products, # 100-106), 100 μ/mL Penicillin-Streptomycin (Gibco™, # 15140122). THP-1 cells were obtained from ATCC and cultured in RPMI 1640 (Thermo Fisher Scientific, #11875-119) supplemented with 10% FBS, and 100 μ/mL Penicillin-Streptomycin.

Mouse HNSCC cell lines MOC1, MOC2, and MOC1-esc1 cells were originally generated in our group and cultured in Iscove’s modified Dulbecco’s medium (Gibco, # 12440053)/ Ham’s Nutrient Mixture F12 (Cytiva, # SH30026.01) at a 2:1 mixture with 5% FBS, 5 ng/mL Epidermal growth factor (MilliporeSigma, # 01-107), 400 ng/mL Hydrocortisone (Sigma-Aldrich, #H0135), 5 mg/mL insulin (Sigma-Aldrich, #I6634) and 100 μ/mL Penicillin-Streptomycin^23,38^.

*Mycoplasma* infection was routinely examined by PCR using the conditioned media after 6 days of culture, using the Mycoplasma Detection kit (ATCC, # 30-1012 K).

### Dissociation of HNSCC patient tumor explants

Fresh HNSCC tumor samples were collected from patients after standard surgery in the Dana-Farber Cancer Institute (DFCI) Head and Neck Surgery department, and processed into tumor explants following the previously established patient-derived organotypic tumor spheroids (PDOTS) protocol^29,30^. First, the HNSCC patient tumors were digested with scissors, then by the Tumor Dissociation Kit (Miltenyi Biotec, # 130-095-929) and a gentleMACS dissociator (Miltenyi Biotec) using the h-TDK-2 program for 15 min. Second, the mixture was filtered through the 100 μm Cell Strainer (Westnet, # 229485) and the tumor explants (above 100 μm) were collected. Third, the tumor explants were cultured in Ultra-Low Attachment 24-Well Plates (Corning, #3473) with 1 mL DMEM containing 10% FBS, 100 U/mL Penicillin-Streptomycin, as well as three antifungals including 2.5 μg/mL Amphotericin B (Sigma-Aldrich, #A2942-100ML), 5 μg/mL Caspofungin diacetate (Sigma-Aldrich, #SML0425-5MG) and 100 μg/mL Primocin (Invivogen, #NC9141851). The tumor explants were stimulated with 50 μM ADU for 24 h. The conditioned media were collected for cytokine profiling.

All patient samples were collected under Dana-Farber/ Harvard Cancer Center institutional review board (IRB) approved protocol #09-472 and studies were performed according to DFCI approved protocol #18-092. Written informed consent was obtained from all patients who donated tumor sample for the study. All patient studies were performed according to the World Medical Association Declaration of Helsinki and IRB-approved protocols. All the tissue samples used were destroyed after the analysis.

### Immunoblotting

Cells were lysed in RIPA Lysis and Extraction Buffer (Thermo Scientific, #89901) containing Protease Inhibitor Cocktail (Roche, #11836170001) and phosphatase inhibitor cocktail tablets (Sigma, #4906845001). 20–50 μg protein samples were loaded for each well. Immunoblotting was performed^18,46^ using the following antibodies: Phospho-STING (Ser366) (Cell Signaling Technology, #50907), STING (Cell Signaling Technology, #13647), IRF3 (Cell Signaling Technology, #11904), phospho-IRF3 (Cell Signaling Technology, # 4947), TBK1 (Cell Signaling Technology, #3504), phospho-TBK1 (Cell Signaling Technology, # 5483), STAT1 (Cell Signaling Technology, #9172), phospho-STAT1 (Cell Signaling Technology, #9167), PTPN2 (Proteintech, # 11214-1-AP), PTPN1 (Abcam,#ab244207), TREX1 (Abcam, #ab185228) and β-Actin (Cell Signaling Technology, #3700). All primary antibodies were used at a 1:1000 dilution. Secondary antibodies (1:5000) were from LICOR Biosciences: IRDye 800CW Goat anti-Rabbit IgG (#926-32211) and IRDye 680LT Goat anti-Mouse IgG (#926-68020). The imaging of blots was acquired using the LICOR Odyssey system.

### Cytokine profiling

Human IFN-β (Thermo Fisher Scientific, #414101), human CXCL10 (R&D systems, # DIP100), human TNF-α (R&D systems, #STA00D), and human IFN-γ (Thermo Fisher Scientific, #SIF50C), as well as mouse IFN-β (R&D systems, # MIFNB0) and mouse CXCL10 (R&D systems, # DY466) ELISAs were performed according to the manufacturer’s protocols.

Luminex assays were performed by the human Cytokine/Chemokine Magnetic Bead Panel (Merck Millipore, #HCYTMAG-60K-PX30) according to the manufacturer’s instructions. Fold change (FC) relative to the corresponding control was calculated and plotted as Log_2_(FC).

### Reagents

The reagents used include Decitabine (Selleckchem, #S1200), Tazemetostat (Selleckchem, #S7128), ADU (Chemietek, #CT-ADUS100), PTPN2/1 inhibitor ABBV-CLS-484 (MedChemExpress, # HY-145923), recombinant human IFN-β protein (R&D systems, # 8499-IF), recombinant human IFN-γ protein (R&D systems, # 285-IF), recombinant mouse IFN-β protein (R&D systems, # 8234-MB), recombinant mouse IFN-γ protein (R&D systems, # 485-MI).

### Immunofluorescence imaging

HSC-2 cells (2 × 10^3^) were grown on 8 chamber cell culture slides (CellTreat, #229168) and were allowed to adhere for 24 h, and then treated with vehicle, AC484 (10 μM), and or IFN-γ (10 μg/mL) as indicated. After 48 h of treatment, cell culture media were aspirated, cells were washed in PBS (×2), fixed in 4% Paraformaldehyde (PFA) for 15 min at room temperature, washed in PBS (×2), and permeabilized with 0.1% v/v Triton-X-100 (Sigma-Aldrich, #93443) for 5 min at room temperature. Permeabilized cells were then washed in PBS (×2) and blocked with a 2% BSA solution (Sigma-Aldrich, #A9576) for 1 h while shaking at room temperature. Blocked cells were then stained with STING antibody (Invitrogen, # PA5-23381) at a 1:200 dilution overnight at 4 °C while shaking. The following day, cells were washed in PBS (x3) and stained with Alexa Fluor 555 goat anti-rabbit secondary antibody (Invitrogen, #A-21428) at a 1:1000 dilution for 2 h at room temperature. Secondary stained cells were then washed in PBS (×5) followed by 70% ethanol and then 100% ethanol. After briefly being allowed to dry, DAPI (4’,6-diamidino-2-phenylindole) mounting media (VectaShield, #H1800) was added and coverslips were applied. The following day, images were acquired using 561 nm and 405 nm lasers on a Zeiss LSM 980 Confocal with Airyscan2 mode and a 25× objective. Acquired images were then analyzed and exported on Zen Blue (version 3.4) microscopy software.

### Quantitative RT-PCR

RNA extraction was performed using RNeasy Mini Kit (Qiagen, # 74106) according to the manufacturer’s protocols. RNA samples (1 μg) were reverse-transcribed using SuperScript® III First-Strand Synthesis SuperMix (Thermo Fisher Scientific, # 1683483). Quantitative real-time PCR was conducted using Power SYBR Green PCR Master Mix (Thermo Fisher Scientific, # 4367659). The sequences of the primers are according to our previous paper^18^, human *STING*: Forward (5′−3′: CCAGAGCACACTCTCCGGTA), Reverse (5′−3′: CGCATTTGGGAGGGAGTAGTA), and the reference gene human *36B4*: Forward (5′−3′: CAGATTGGCTACCCAACTGTT), Reverse (5′−3′: GGAAGGTGTAATCCGTCTCCA). Values represent the average of technical triplicates.

### CRISPR/Cas9 system

sgRNA target sequences for CRISPR interference were designed by the sgRNA designer (http://portals.broadinstitute.org/gpp/public/analysis-tools/sgrna-design). The scramble sgRNA indicated the non-targeting sgRNA from the Get-go library v2. sgRNAs target human TREX1 and PTPN2 were cloned into the lentiCRISPRv2 puro plasmid, and sgRNAs target human STING and PTPN1 were cloned into the lentiCRISPR v2-Blast plasmid. sgRNA target sequences (5′−3′) are according to our previous paper^18,20,21^,

human *TREX1* sgRNA2 (GTCCCCTCCAGACTCGCACA),

human *TREX1* sgRNA3 (TCTGGATGGTGCCTTCTGTG),

human *STAT1* sgRNA (TGCTGGCACCAGAACGAATG),

human *STING* sgRNA1 (TCAGCCATACTCAGGTTATC),

human *STING* sgRNA2 (GCAGGCACTCAGCAGAACCA),

human *STING* sgRNA4 (GGTACCGGGGCAGCTACTGG).

as well as generated in this paper,

human *PTPN2* sgRNA1 (GCACTACAGTGGATCACCGC),

human *PTPN2* sgRNA2 (GGGTCTGAATAAGACCCATT),

human *PTPN2* sgRNA3 (TGTCATGCTGAACCGCATTG),

human *PTPN1* sgRNA2 (GAAGCTTGGCCACTCTACAT) and

human *PTPN1* sgRNA4 (GTACAGTGCGACAGCTAGAAT).

### Generation of lentivirus

5 × 10^6^ HEK-293T cells were pre-plated onto a 60-mm dish overnight and transfected with Opti-MEM (Thermo Fisher Scientific, # 51985034), X-tremeGENE HP DNA Transfection Reagent (Roche, #06366236001), 4 μg of lentivirus-based expression vectors (pCRISPR-v2 sgRNAs), 2 μg of pCMV-dR8.91 and 2 μg of pCMV-VSV-G. Culture media was replaced 18 h later after transfection. After another 48 h, the media containing lentivirus particles were collected and filtered through the 0.45 μm Syringe Filters. The infected cells were continuously under puromycin (1 μg/mL) selection during subculture. For STING overexpression in HSC-2 cells, we used the plx304-hSTING plasmid from our previous paper^14^.

### PBMC-derived NK cell isolation and expansion

Peripheral blood mononuclear cells (PBMCs) were isolated using BD Vacutainer® CPT™ (BD Biosciences, # 362760) from apheresis leukoreduction collars which were collected by Crimson Core at Brigham and Women’s Hospital as fresh byproducts of platelet donation from anonymized healthy donors (domestic study ID# T0847), under the protocol Mass General Brigham IRB# 2005P001742 and the protocol DFCI-IRB# NHSR#428198. NK cells were then isolated from the PBMCs using the EasySep™ Human NK Cell Isolation Kit (STEMCELL, # 17955) following the manufacturer’s instructions. The NK cells were cultured in NK MACS® Medium (Miltenyi Biotec, #130-114-429) supplemented with 5% human serum (Sigma-Aldrich, # H5667), and 500 IU/mL recombinant human IL-2 (R&D Systems, # 202-IL). The medium was refreshed every 3 days. The cultured NK cells were used for subsequent experiments during day 8 and day 35.

### Flow cytometric cell viability assay

HSC-2 cells (2 × 10^5^/well) were seeded in 6-well plates for 24 h. These cells are treated with AC484 (10 μM) and human IFN-γ (10 ng/mL) for 72 h and then stained with Annexin V (BioLegend, #640912) and Helix NP™ Green (BioLegend, #425303) according to the manufacturer’s protocol. Antibodies were used at a 1:50 dilution.

For HSC-2 and NK cells co-culture assay, AC484 (10 μM), or NK cells (40 × 10^3^) were added at Effector cell: Target cell ratio of 1:2 on the next day. The culture media was a 1:1 mixture of NK MACS® Medium and DMEM medium containing 500 IU/mL IL-2. 72 h later, cells were collected and stained with Annexin V and Helix NP™ Green. The sample was examined by the BD LSRFortessa™ Cell Analyzer. FlowJo v10 software was used to perform the raw data analysis.

### CellTiter-Glo luminescent cell viability assay

1 × 10^4^ HSC-2 cells per well were seeded in a 96-well Microplate (Corning, # CLS3917) and were allowed to adhere for 24 h. Next, HSC-2 cells were pre-treated with AC484 (10 μM) and human IFN-γ (10 ng/mL) for 24 h to increase STING. Last, add ADU (50 μM) for an additional 24 h of treatment.

Similarly, for HSC-2 cells and NK cells co-culture assay, 10 × 10^3^ HSC-2 cells per well were seeded in a 96-well Microplate and were allowed to adhere for 24 h. The culture media was a 1:1 mixture of NK MACS® Medium and DMEM medium containing 500 IU/mL IL-2. Next, HSC-2 cells were pre-treated with AC484 (10 μM) and PBMC-derived NK cells (10 × 10^3^) for 24 h to increase STING. Last, adding ADU (50 μM) for an additional 24 h treatment. The plates were read on a Tecan Infinite Mplex Microplate Reader according to the manufacturer’s protocols (Promega, #G7571). All conditions were tested in biological triplicates for each independent experiment. Three independent experiments. Values were normalized to vehicle-treated cells. Triplicates or quadruplicates for each condition.

### In vivo studies

Research complies with relevant ethical regulations. Specifically, studies using the MOC1-esc1 tumor model were conducted at Broad Institute, under protocol # 0110-08-16-3, approved by the Institutional Animal Care and Use Committee (IACUC) of the Broad Institute. Study using the MOC2 tumor model study was conducted at DFCI, under protocol # 04-111, approved by the IACUC of DFCI. 6~8 weeks old female C57BL/6 mice or immunodeficient female NOD.Cg-PrkdcscidIl2rgtm1Wjl/SzJ (NSG) mice (Strain # 005557) ordered from the Jackson Laboratory. As MOC models are female C57BL/6 derived, only female mice were included in the studies. All mice were housed in a specific pathogen-free animal facility. MOC1-esc1 cells and MOC2 that reached about 70~80% confluence in vitro were collected, washed with PBS twice, and then resuspended in PBS. 3 × 10^6^ MOC1-esc1 cells or 0.1 × 10^6^ MOC2 cells in 70 μL PBS, mixed with 30 μL Matrigel (Corning, #356231) on ice, were subcutaneously injected into one flank of each mouse. Mice were randomized to treatment group (5~7 mice for each group) on day 7. One dose of ADU (20 μg ADU dissolved in 50 μL PBS) was given by intratumoral injection on day 7. AC484 was dissolved in DMSO and administered as a solution in 0.5% Hydroxypropyl methylcellulose (HPMC) by oral gavage (50 mg/kg) daily for three consecutive weeks beginning on day 7. For Immunohistochemistry (IHC) of STING and p-TBK1, as well as profiling tumor infiltrating immune cells by flow cytometry, tumor samples were collected on day 12. Animals were euthanized when tumor volume reached 2000 mm^3^. The length (L) and width (W) of the tumor were measured twice per week by digital caliper, and the tumor volume was calculated as the Volume = 0.5 × L × W × W.

### Immunohistochemistry

MOC1-esc1 tumor tissues were harvested on day 12 and preserved by Formalin-Fixed Paraffin-Embedded (FFPE). Immunohistochemistry was conducted on the Leica Bond III automated staining platform at the Department of Pathology at Brigham and Women’s Hospital. The slides were stained with STING (Cell Signaling Technology, #13647) and phospho-TBK1 (Cell Signaling Technology, # 5483). The images for each tumor tissue slide were required by using the Akoya Biosciences Vectra Polaris™ Automated Quantitative Pathology Imaging System at 20× magnification. Tumor cell STING IHC staining was quantified by an board-certified pathologist (N.R.M.) by H-score in five randomly selected regions of interest (ROI). These IHC images were captured by Phenochart Whole Slide Viewer (Akoya Biosciences).

### Immune profiling by flow cytometry

For profiling the tumor infiltration immune cells in MOC1-esc1 tumor tissue that treated with ADU (20 μg; one dose on day 7) and AC484 (50 mg/kg; once per day for five consecutive days), whole tumor tissue was collected on day 12. The tumors were dissociated into single cells through the kit from Miltenyi Biotec (#130-096-730) according to the manufacturer’s instructions. The following flow cytometry antibodies were used: Live/dead IR (Invitrogen, #L10119), CD45-BUV395 (BD Bioscience, # 564279), TCRβ-FITC (BioLegend, #109205), NK1.1-Brilliant Violet 421 (BioLegend, # 156537), CD4-PE/Dazzle™ 594 (BioLegend, #100456), CD8a-Alexa Fluor® 700 (BioLegend, #155022), F4/80-APC (Biolegend, #123116), CD11b-PE (Biolegend, #101208), CD11c-FITC (Biolegend, #117305), CD103-PE/Dazzle™ 594 (Biolegend, #121430), MHC class II -PerCP/Cyanine5.5 (Biolegend, # 116416).

For intracellular staining, fixation and permeabilization of cells were by the Foxp3 / Transcription Factor Staining Buffer Set (Invitrogen, # 00-5523-00) according to the manufacturer’s protocol. Foxp3-APC (Invitrogen, #17-5773-82) and Perforin-PE (BioLegend, #154306) were used. FlowJo v10 software was used to perform the raw data analysis. Antibodies were used at a 1:50 dilution.

### In vivo NK cell depletion

200 μg of anti-mouse NK1.1 antibody (bioxcell, clone PK136) was administered by intraperitoneal injections on days −1, 2, 6, and 10. Equal amounts of IgG isotype antibodies were given as control. NK cell depletion efficiency was validated by flow cytometry from collected peripheral blood on day 12.

### NK cell–infiltration assay in the 3D microfluidics device

HSC-2 cells (0.5 × 10^6^ cells per well of 6-well plate) were loaded into Ultra-Low Attachment plate (Corning, #3471) overnight to form tumor spheroids. Next, HSC-2 tumor spheroids (80 × 10^3^) were collected and resuspended with Collagen (3.5 mg/L, pH = 7.0) (Corning, #354236) on ice. The mixture was loaded into the AIM 3D microfluidic chip (10 µL per well). Leave the microfluidic chip in 37° incubator for 25 min for collagen polymerization. Meanwhile, NK cells (20 × 10^3^ in 10 µL culture medium) labeled with CellTrace™ Yellow (ThermoFisher,#C34567) according to the manufacture’s protocol, and then were gently loaded in the left side channel of the 3D microfluidic device. 150 µL culture media (DMEM media: NK cell media=1:1) containing AC484 (10 µM) and IL-2 (500 IU/mL) were added to each well on day 0. On day 2, additional 150 µL culture media (DMEM media: NK cell media=1:1) containing AC484 (10 µM), IL-2 (500 IU/mL) and ADU (100 µM) were added to each well. On Day 4, HSC-2 tumor spheroids were stained with DNA marker Hoechst 33342 (Invitrogen, #H3570) (used at a 1:800 dilution) and dead cell marker DRAQ7 (Biolegend,#424001) (used at a 1:100 dilution) according to the manufacture’s protocol.

The immunofluorescence images were captured by a Nikon Eclipse 80i fluorescence microscope equipped with Z-stack (Prior) and CoolSNAP CCD camera (Roper Scientific). Image capture and analysis were performed using NIS-ElementsARsoftware package. Viability of HSC-2 tumor spheroids was quantified as below: (1- Binary area of DRAQ7/ Binary area of Hoechst 33342) x100%. Biological triplicates for each group.

### Reporting summary

Further information on research design is available in the Nature Portfolio Reporting Summary linked to this article.

## Supplementary information

Supplementary Information

Reporting Summary

Transparent Peer Review file

## Source data

Source data

## Data Availability

All the data supporting the findings of this study are available within the Article, its Supplementary Information and/or Source Data file. Source data are provided with this paper.
