# Supplementary material for: PTPN2 inhibition unleashes response to STING agonism in head and neck squamous cell cancer
Source: Nat Commun. 2026 May 2;17:5958. doi: 10.1038/s41467-026-72372-1 (PMC13342608; doi:10.1038/s41467-026-72372-1)
Supplement: Supplementary file 1 — Supplementary Information [file 41467_2026_72372_MOESM1_ESM.pdf]

Supplementary Information

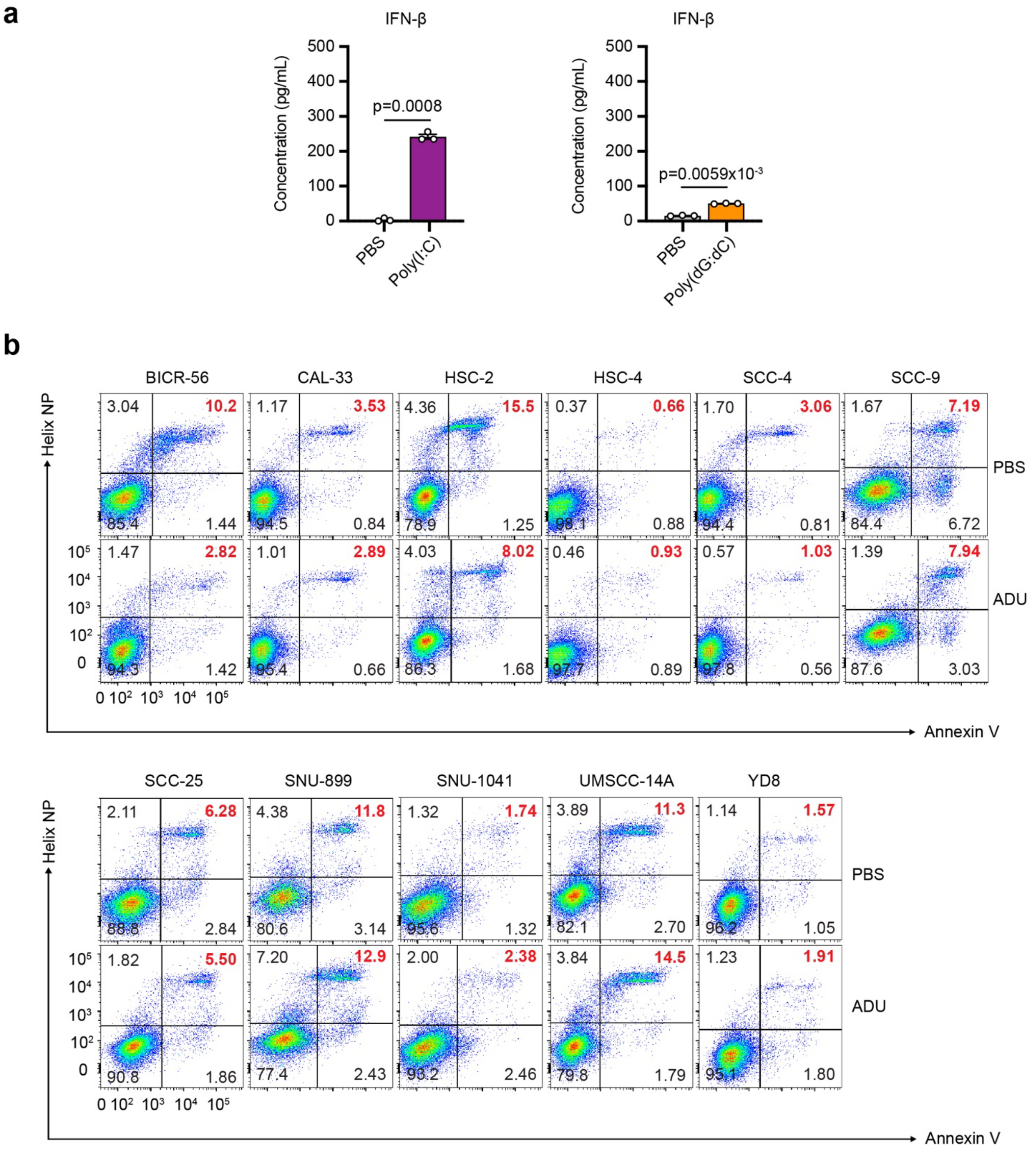

Supplementary Fig 1. ADU does not induce apoptosis of multiple human HNSCC cell lines (related with Fig.1).

a, ELISA of IFN-β in conditioned media derived from HSC-2 cells, with or without Poly(I:C) (10 μg/mL) and Poly(dG:dC) (0.5 μg/mL) treatment for 24 hours (24H) (three independent biological samples).

b, Representative flow cytometry plots were shown. HNSCC cells were treated with ADU (50 μM) for 72H and stained with Annexin V and Helix NP. The percentage of dead cells indicates the proportion of Annexin V+ and/or Helix NP+ stained cells (three independent biological samples).

Data in **a** was calculated by unpaired t test. Data are represented as mean ± SEM. Source data are provided as a Source Data file.

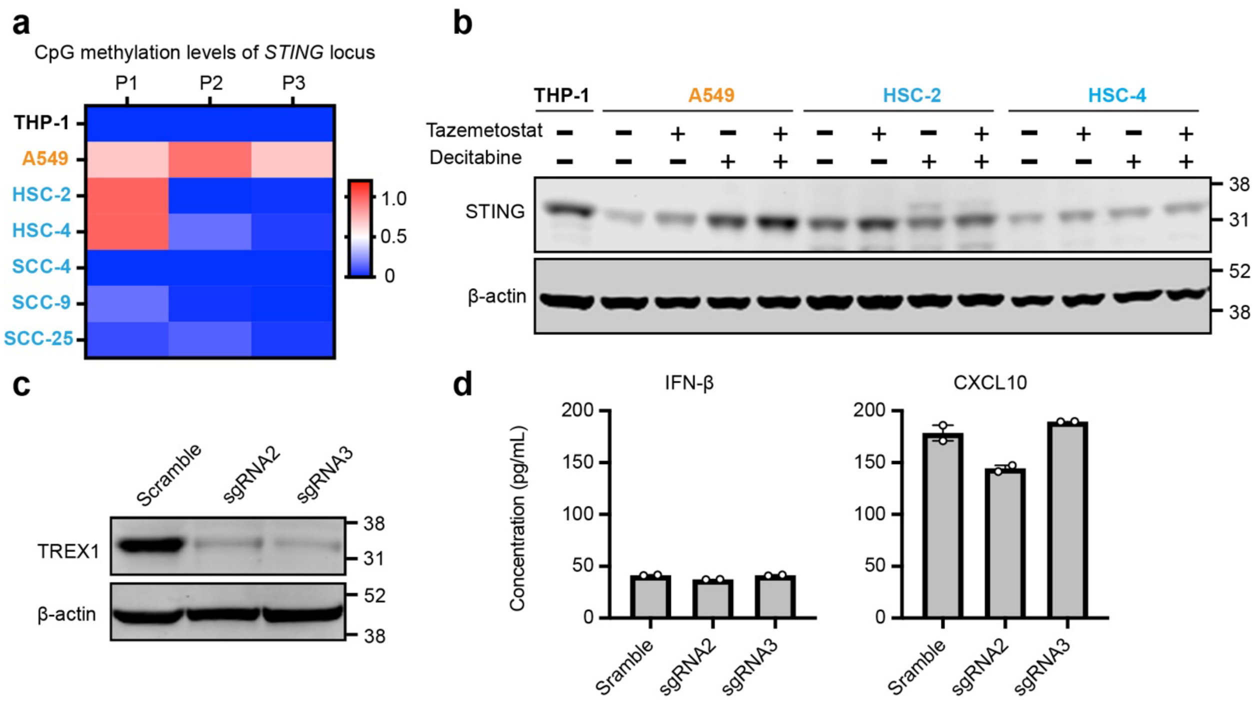

Supplementary Fig 2. Neither DNMT inhibitor/EZH2 inhibitor treatment, nor deletion of TREX1 increases STING in HSC-2 cells (related with Fig.3).

a, Heatmap of the CpG methylation levels at three positions (P1, P2, and P3) at *STING* locus from CCLE repository.

b, Immunoblot of STING from HSC-2 cells treated with DNMTs inhibitor (decitabine; 100 nM) and EZH2 inhibitor (Tazemetostat; 5 μM) for 5 days. Fresh media /drugs were replaced on day 3. Data are representative of one independent experiment.

c, Immunoblot of TREX1 in HSC-2 cells transduced with the indicated vectors. sgRNA2 and sgRNA3 (two distinctive single guide RNA targeting TREX1). Data are representative of one independent experiment.

d, ELISA of IFN-β and CXCL10 in conditioned media derived from (**c**) (two independent biological samples).

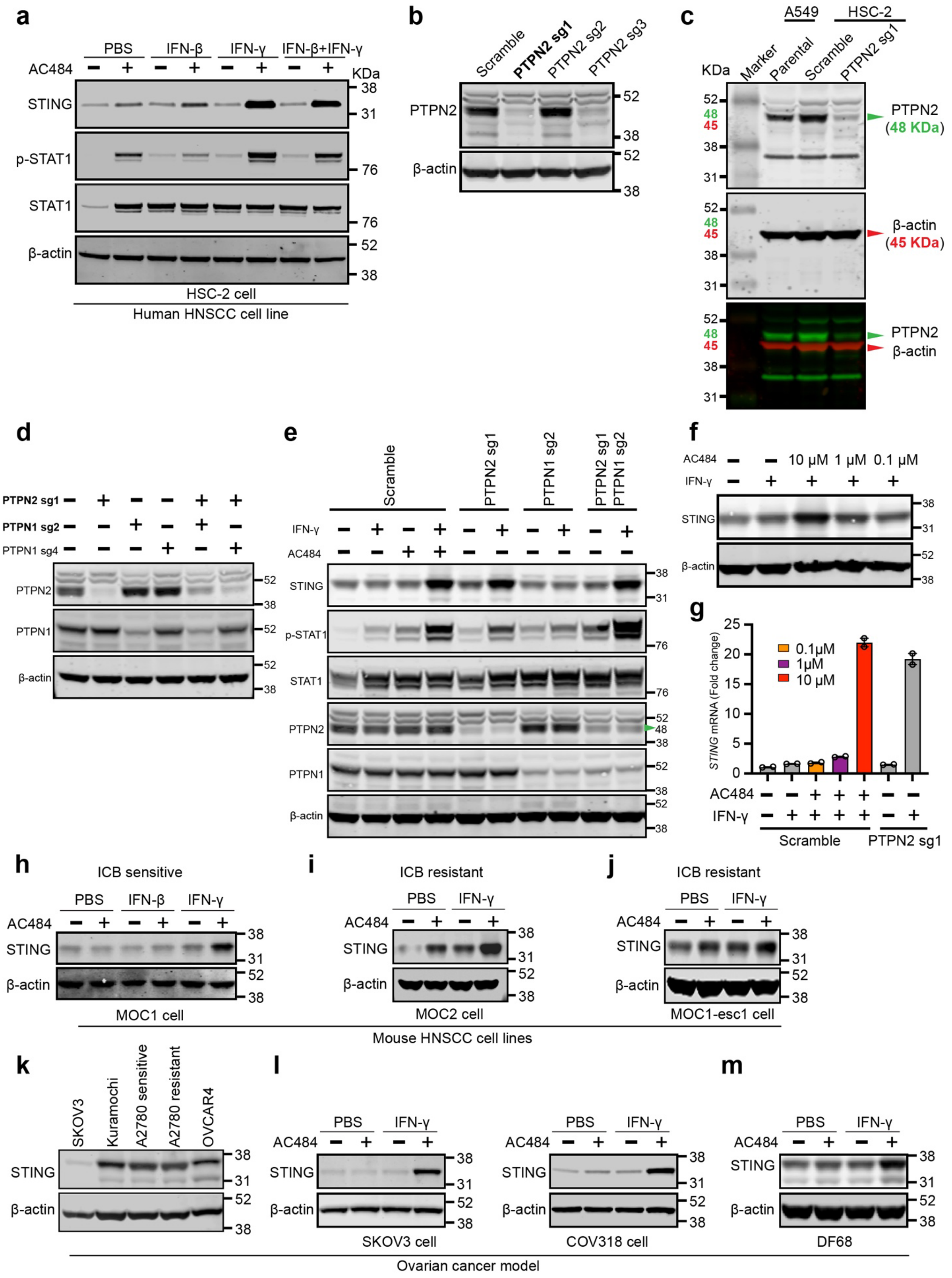
a
PBS
IFN-β
IFN-γ
IFN-β+IFN-γ
AC484
KDa
STING
p-STAT1
STAT1
β-actin
HSC-2 cell
Human HNSCC cell line
b
Scramble
PTPN2 sg1
PTPN2 sg2
PTPN2 sg3
PTPN2
β-actin
c
A549
HSC-2
KDa
Marker
Parental
Scramble
PTPN2 sg1
PTPN2 (48 KDa)
β-actin (45 KDa)
PTPN2
β-actin
d
PTPN2 sg1
PTPN1 sg2
PTPN1 sg4
PTPN2
PTPN1
β-actin
e
Scramble
PTPN2 sg1
PTPN1 sg2
PTPN2 sg1 PTPN1 sg2
IFN-γ
AC484
STING
p-STAT1
STAT1
PTPN2
PTPN1
β-actin
f
AC484
10 μM 1 μM 0.1 μM
IFN-γ
STING
β-actin
g
STING mRNA (Fold change)
0.1μM
1μM
10 μM
AC484
IFN-γ
Scramble
PTPN2 sg1
h
ICB sensitive
PBS
IFN-β
IFN-γ
AC484
STING
β-actin
MOC1 cell
i
ICB resistant
PBS
IFN-γ
AC484
STING
β-actin
MOC2 cell
j
ICB resistant
PBS
IFN-γ
AC484
STING
β-actin
MOC1-esc1 cell
Mouse HNSCC cell lines
k
SKOV3
Kuramochi
A2780 sensitive
A2780 resistant
OVCAR4
STING
β-actin
l
PBS
IFN-γ
AC484
STING
β-actin
SKOV3 cell
COV318 cell
m
PBS
IFN-γ
AC484
STING
β-actin
DF68
Ovarian cancer model

29

Supplementary Fig 3. AC484 plus IFN-γ, but not IFN-β, increases tumor cell STING (related with Fig.3).

a, Immunoblot of HSC-2 cells treated with human IFN-β (10 ng/mL) and IFN-γ (10 ng/mL), with or without AC484 (10 μM) for 48 hours. Data are representative of one independent experiment.

b, Immunoblot of PTPN2 proteins in HSC-2 cells transduced with the indicated vectors.

c, Immunoblot of PTPN2 proteins in parental A549 cells and HSC-2 cells transduced with the indicated vectors. Data are representative of one independent experiment.

d, Immunoblot of PTPN2 and PTPN1 proteins in HSC-2 cells transduced with the indicated vectors. Data are representative of one independent experiment.

e, Immunoblot of indicated proteins in HSC-2 cells transduced with the indicated vectors and treated with AC484 (10 μM) and IFN-γ (10 ng/mL) for 48 hours.

f, Immunoblot of STING in HSC-2 cells treated with AC484 (10 μM; 1 μM; 0.1 μM) and IFN-γ (10 ng/mL) for 48 hours. Data are representative of one independent experiment.

g, qPCR assay of STING in indicated cells treated with AC484 (10 μM; 1 μM; 0.1 μM) and IFN-γ (10 ng/mL) for 24 hours (Two independent biological samples).

h, Immunoblot of MOC1 cells treated with mouse IFN-β (1 ng/mL) and IFN-γ (1 ng/mL), with or without AC484 (1 μM) for 48 hours. Data are representative of one independent experiment.

i, Immunoblot of MOC2 cells treated with mouse IFN-γ (1 ng/mL), with or without AC484 (1 μM) for 48 hours. Data are representative of one independent experiment.

j, Immunoblot of MOC1-esc1 cells treated with mouse IFN-γ (1 ng/mL), with or without AC484 (1 μM) for 12 hours. Data are representative of one independent experiment.

k, Immunoblot of endogenous STING level in multiple ovarian cancer cell lines. Data are representative of one independent experiment.

l, Immunoblot of indicated proteins in human ovarian cancer SKOV3 and COV318 cells treated with IFN-γ (10 ng/mL) and AC484 (10 μM) for 48 hours. Data are representative of one independent experiment.

m, Immunoblot of indicated proteins from DF68 treated with IFN-γ (10 ng/mL) and AC484 (10 μM) for 48 hours. Data are representative of one independent experiment.

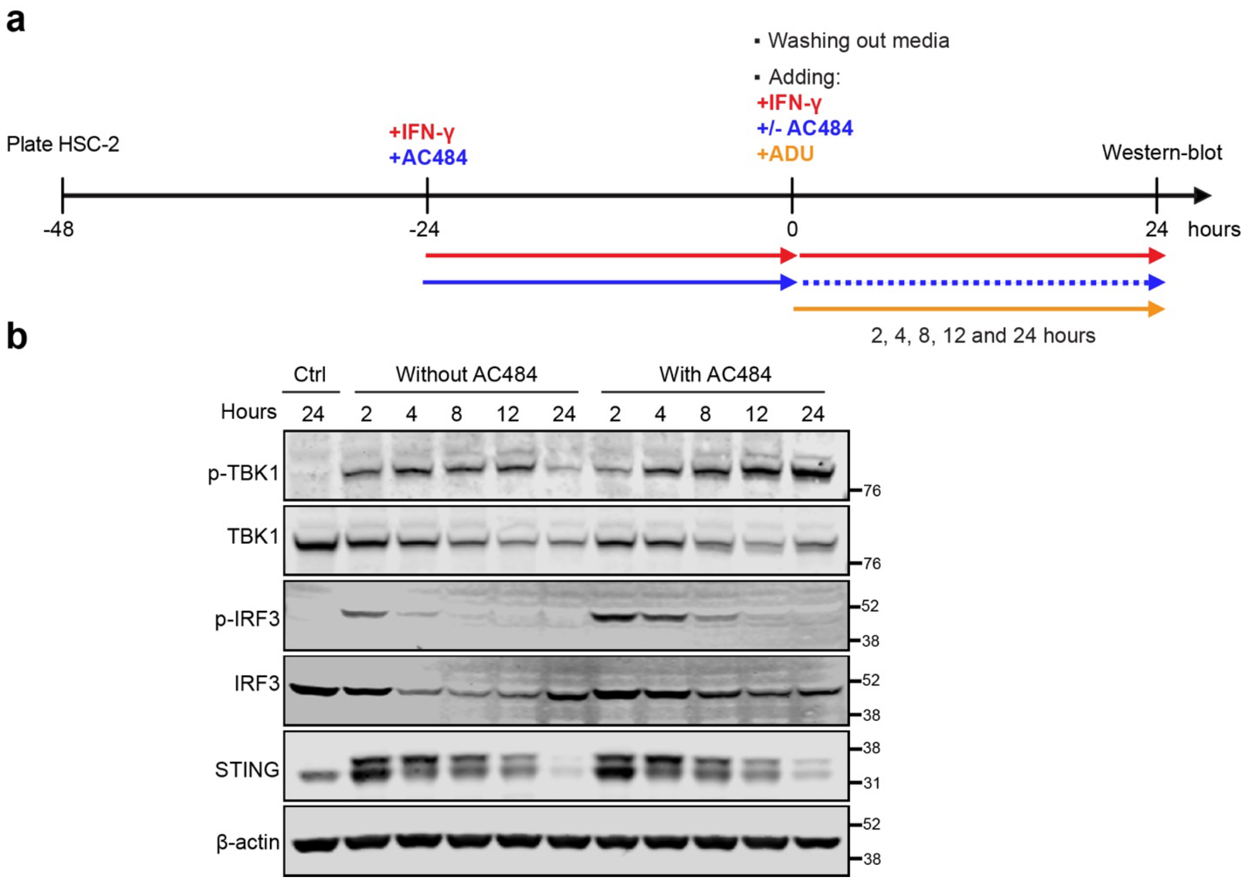

Supplementary Fig 4. AC484 does not delay ADU induced STING degradation (related with Fig.4).

a, Schematic of sequential drug treatment. HSC-2 cells were pretreated with IFN-γ (10 ng/mL) and AC484 (10 μM) for 24 hours, and AC484 was deprived by washing twice with

PBS. Fresh media containing IFN-γ (10 ng/mL) and ADU (50 μM) was added for an additional 2, 4, 8, 12, and 24 hours, with or without AC484 (10 μM).

b, Immunoblot of indicated proteins from HSC-2 cells treated with drugs as shown in (**a**). Data are representative of one independent experiment.

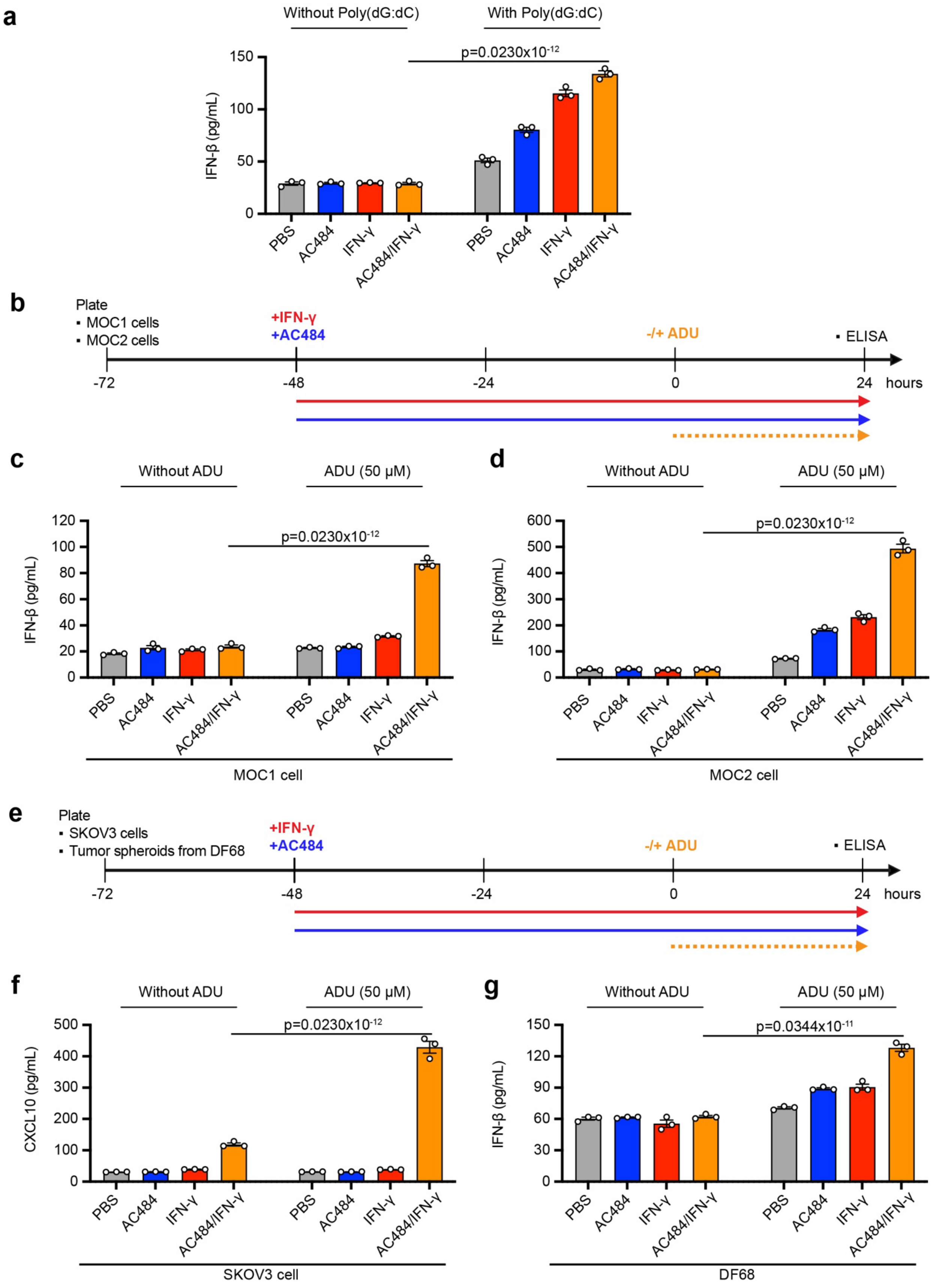
a
Without Poly(dG:dC)
With Poly(dG:dC)
p=0.0230x10-12
IFN-β (pg/mL)
150
100
50
0
PBS
AC484
IFN-γ
AC484/IFN-γ
PBS
AC484
IFN-γ
AC484/IFN-γ
b
Plate
▪ MOC1 cells
▪ MOC2 cells
+IFN-γ
+AC484
-/+ ADU
▪ ELISA
-72
-48
-24
0
24
hours
c
Without ADU
ADU (50 μM)
p=0.0230x10-12
IFN-β (pg/mL)
120
100
80
60
40
20
0
PBS
AC484
IFN-γ
AC484/IFN-γ
PBS
AC484
IFN-γ
AC484/IFN-γ
MOC1 cell
d
Without ADU
ADU (50 μM)
p=0.0230x10-12
IFN-β (pg/mL)
600
500
400
300
200
100
0
PBS
AC484
IFN-γ
AC484/IFN-γ
PBS
AC484
IFN-γ
AC484/IFN-γ
MOC2 cell
e
Plate
▪ SKOV3 cells
▪ Tumor spheroids from DF68
+IFN-γ
+AC484
-/+ ADU
▪ ELISA
-72
-48
-24
0
24
hours
f
Without ADU
ADU (50 μM)
p=0.0230x10-12
CXCL10 (pg/mL)
500
400
300
200
100
0
PBS
AC484
IFN-γ
AC484/IFN-γ
PBS
AC484
IFN-γ
AC484/IFN-γ
SKOV3 cell
g
Without ADU
ADU (50 μM)
p=0.0344x10-11
IFN-β (pg/mL)
150
120
90
60
30
0
PBS
AC484
IFN-γ
AC484/IFN-γ
PBS
AC484
IFN-γ
AC484/IFN-γ
DF68

Supplementary Fig 5. Increased STING restores ADU sensitivity in mouse HNSCC and ovarian cancer cells (related with Fig.4).

a, ELISA for IFN-β derived from conditioned media from HSC-2 cells that were pretreated with IFN-γ (10 ng/mL) and AC484 (10 μM) for 24 hours, and then poly(dG:dC) (0.5 μg/mL) for additional 24 hours (three independent biological samples).

b, Schematic of sequential drug treatment. MOC1 and MOC2 cells were pretreated with mouse IFN-γ (1 ng/mL) and AC484 (1 μM) for 48 hours, and ADU (50 μM) for additional 24 hours.

c, d ELISA for mouse IFN-β derived from conditioned media of MOC1 and MOC2 cells treated as in (**b**) (three independent biological samples).

e, Schematic of sequential drug treatment. SKOV3 cells and DF68 were pretreated with IFN-γ (10 ng/mL) and AC484 (10 μM) for 48 hours, and ADU (50 μM) for additional 24 hours.

f, g ELISA for CXCL10 and IFN-β derived from conditioned media of SKOV3 cells and DF68 that were treated as (**e**) (three independent biological samples).

Data in **a**, **c**, **d**, **f** and **g** were calculated by two-way ANOVA followed by Tukey's multiple comparisons test. Data are represented as mean ± SEM. Source data are provided as a Source Data file.

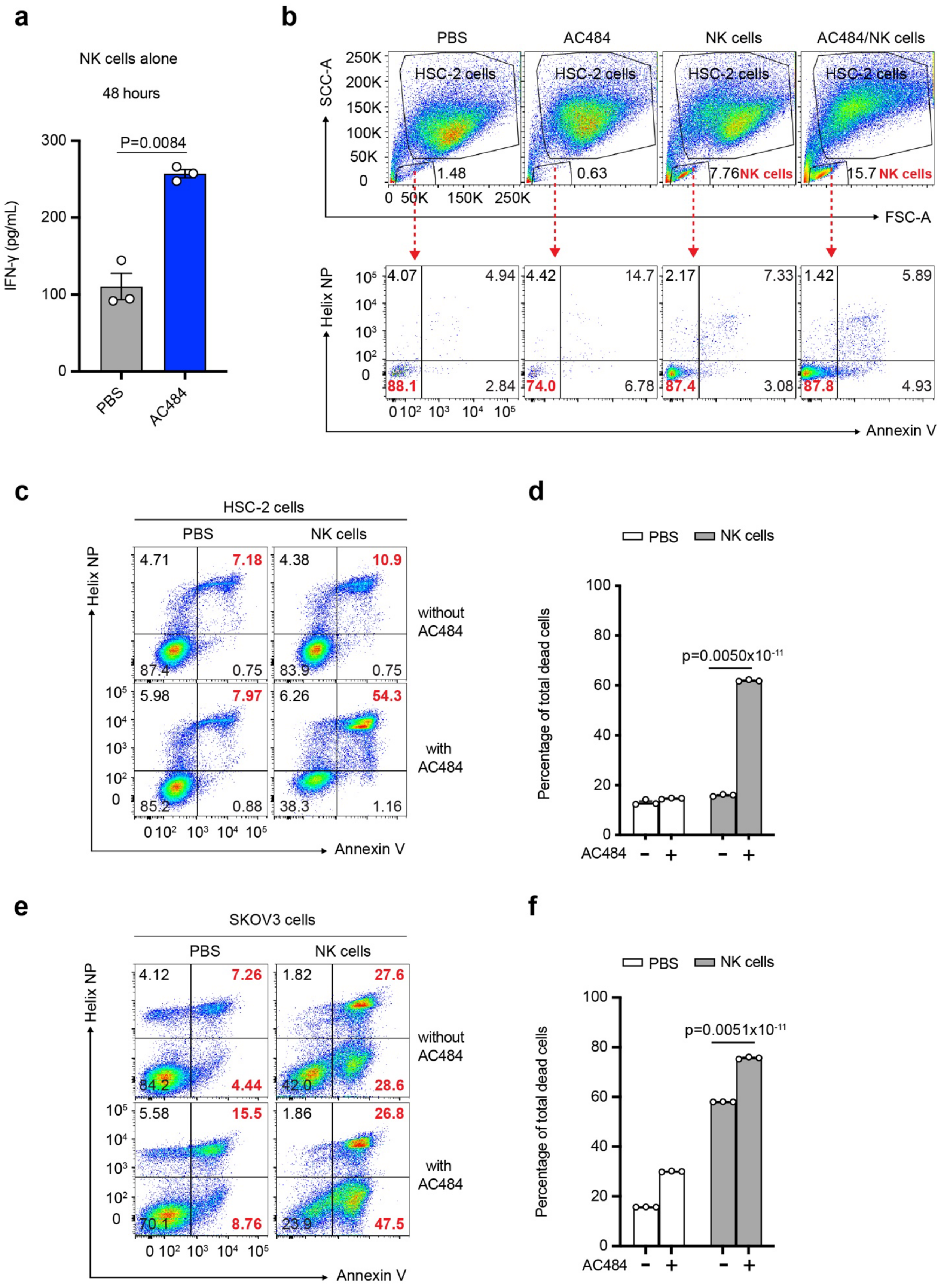
a
NK cells alone
48 hours
P=0.0084
IFN-γ (pg/mL)
PBS
AC484
b
PBS
AC484
NK cells
AC484/NK cells
HSC-2 cells
SCC-A
FSC-A
NK cells
Helix NP
Annexin V
c
HSC-2 cells
without AC484
with AC484
d
PBS
NK cells
Percentage of total dead cells
p=0.0050x10-11
AC484
e
SKOV3 cells
f
p=0.0051x10-11

93

Supplementary Fig 6. AC484 enhances NK cell killing function (related with Fig.5).

a, ELISA for IFN-γ derived from conditioned media from NK cells treated with AC484 (10 μM) for 48 hours (three independent biological samples).

b, Flow cytometric analysis of Annexin V and Helix NP of NK cells in the co-culture assay. PBMC-derived NK cells (10K) and HSC-2 cells (100K) were co-cultured at 1:10, with or without AC484 (10 μM) for 72 hours (three independent biological samples).

c, Flow cytometric analysis of Annexin V and Helix NP of HSC-2 cells gated from (**a**) (three biological replicates).

d, Quantification of the percentage of dead HSC-2 cells, indicated the proportion of Annexin V+ and/or Helix NP+ stained cells (three independent biological samples).

e, Flow cytometric analysis of Annexin V and Helix NP of SKOV3 cells in the co-culture assay. PBMC-derived NK cells (50K) and SKOV3 cells (100K) were co-cultured at 1:2, with or without AC484 (10 μM) for 5 days (three independent biological samples).

f, Quantification of the percentage of dead SKOV3 cells indicated the proportion of Annexin V+ and/or Helix NP+ stained cells (three independent biological samples).

Data in **a** was calculated by one-way ANOVA followed by Tukey's multiple comparisons test. Data in **d** and **f** were calculated by two-way ANOVA followed by Tukey's multiple comparisons test. Data are represented as mean ± SEM. Source data are provided as a Source Data file.

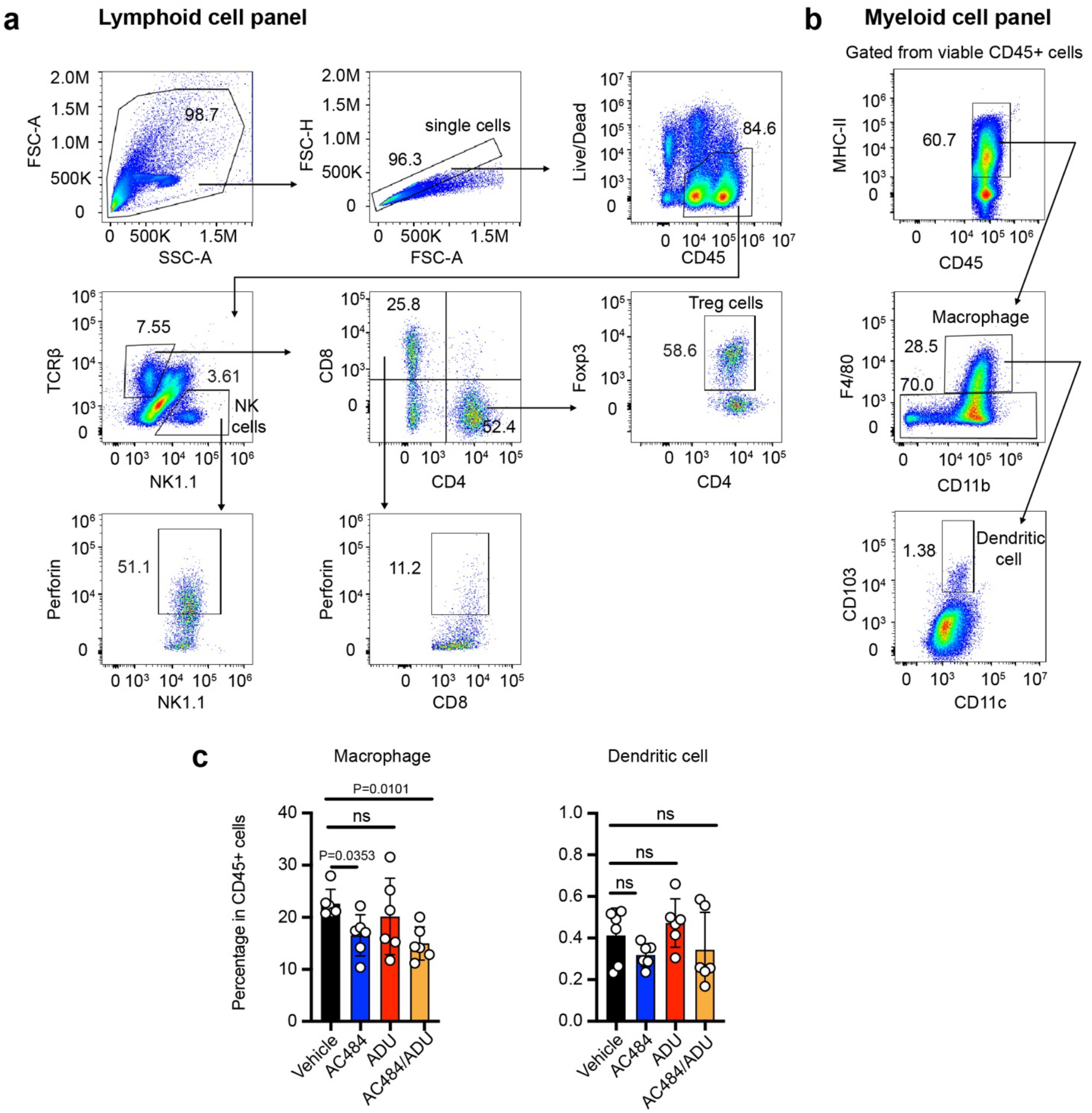

Supplementary Fig 7. Gating strategy of flow cytometric analysis of tumor infiltrating immune cells in MOC1-esc1 TME (related with Fig. 7).

a, Gating strategy of the lymphoid cells in the TME on day 12.

b, Gating strategy of the myeloid cells in the TME on day 12.

c, The percentage of macrophage (MHC-II$^+$F4/80$^+$CD11b$^+$) and dendritic cells (MHC-II$^+$F4/80$^-$CD11b$^-$ CD103$^+$CD11c$^+$) in CD45+ cells were shown (n= 6 mice for each group).

Data in **c** was calculated by one-way ANOVA followed by uncorrected Fisher's Least Significant Difference (LSD) test. Data are represented as mean ± SEM. NS, nonsignificant. Source data are provided as a Source Data file.

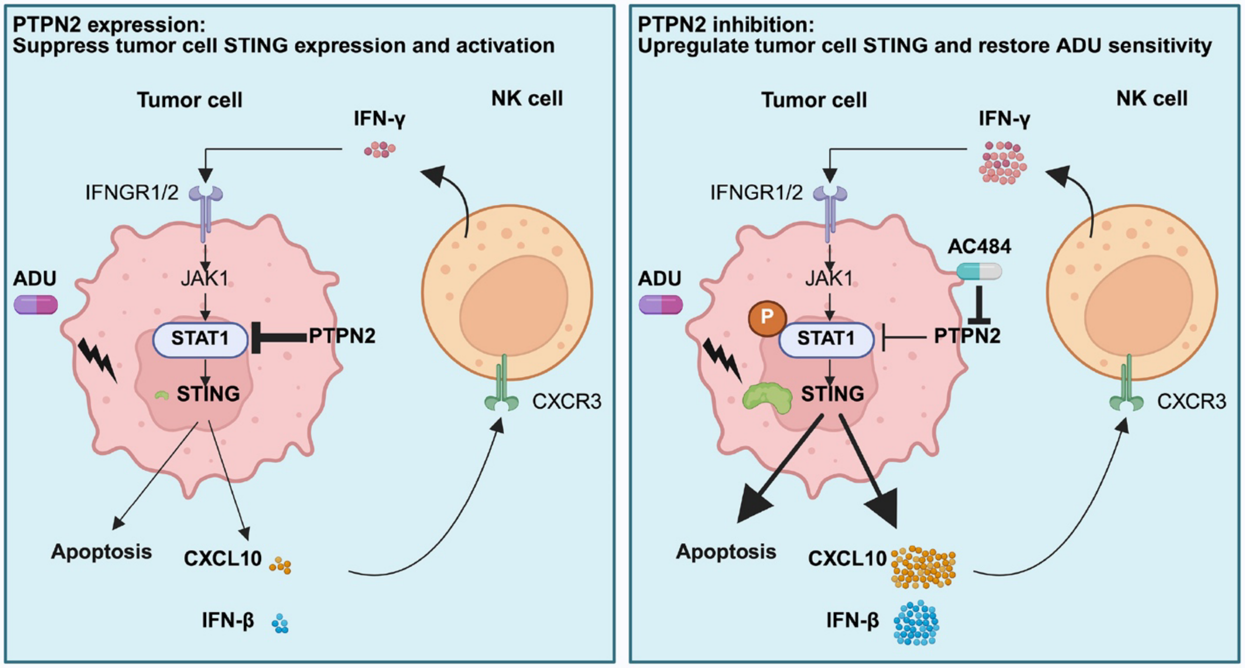

Supplementary Fig 8. HNSCC tumor cells expressed low STING and showed no response to STING agonist ADU. Mechanistically, PTPN2 suppressed STING induction by IFN-STAT1 signaling. The PTPN2/1 inhibitor AC484 plus NK cell derived IFN-γ efficiently increases STING by enhancing p-STAT1, which restores tumor cell sensitivity to ADU. Created in BioRender. Campisi, M. (2026) https://BioRender.com/ l5y0ezr
