# Supplementary material for: PTPN2 inhibition unleashes response to STING agonism in head and neck squamous cell cancer
Source: Nat Commun. 2026 May 2;17:5958. doi: 10.1038/s41467-026-72372-1 (PMC13342608; doi:10.1038/s41467-026-72372-1)
Supplement: Supplementary file 2 — Reporting Summary [file 41467_2026_72372_MOESM2_ESM.pdf]

nature portfolio

Corresponding author(s): Robert T. Manguso, Ravindra Uppaluri & David A. Barbie

Last updated by author(s): Mar 25, 2026

Reporting Summary

Nature Portfolio wishes to improve the reproducibility of the work that we publish. This form provides structure for consistency and transparency in reporting. For further information on Nature Portfolio policies, see our Editorial Policies and the Editorial Policy Checklist.

Statistics

For all statistical analyses, confirm that the following items are present in the figure legend, table legend, main text, or Methods section.

n/a	Confirmed	
☐	☒	The exact sample size (*n*) for each experimental group/condition, given as a discrete number and unit of measurement
☐	☒	A statement on whether measurements were taken from distinct samples or whether the same sample was measured repeatedly
☐	☒	The statistical test(s) used AND whether they are one- or two-sided<br>*Only common tests should be described solely by name; describe more complex techniques in the Methods section.*
☒	☐	A description of all covariates tested
☐	☒	A description of any assumptions or corrections, such as tests of normality and adjustment for multiple comparisons
☐	☒	A full description of the statistical parameters including central tendency (e.g. means) or other basic estimates (e.g. regression coefficient) AND variation (e.g. standard deviation) or associated estimates of uncertainty (e.g. confidence intervals)
☐	☒	For null hypothesis testing, the test statistic (e.g. *F*, *t*, *r*) with confidence intervals, effect sizes, degrees of freedom and *P* value noted<br>*Give P values as exact values whenever suitable.*
☒	☐	For Bayesian analysis, information on the choice of priors and Markov chain Monte Carlo settings
☒	☐	For hierarchical and complex designs, identification of the appropriate level for tests and full reporting of outcomes
☒	☐	Estimates of effect sizes (e.g. Cohen's *d*, Pearson's *r*), indicating how they were calculated

Our web collection on statistics for biologists contains articles on many of the points above.

Software and code

Policy information about availability of computer code

Data collection	ELISA data were collected by Magellan Standard v7.2 Software (Tecan).<br>qPCR data were collected by CFX Maestro Software (4.1.2433.1219).
Data analysis	GraphPad Prism (v10), Microsoft Excel(v15), FlowJo(v10)

For manuscripts utilizing custom algorithms or software that are central to the research but not yet described in published literature, software must be made available to editors and reviewers. We strongly encourage code deposition in a community repository (e.g. GitHub). See the Nature Portfolio guidelines for submitting code & software for further information.

Data

Policy information about availability of data

All manuscripts must include a data availability statement. This statement should provide the following information, where applicable:

- Accession codes, unique identifiers, or web links for publicly available datasets
- A description of any restrictions on data availability
- For clinical datasets or third party data, please ensure that the statement adheres to our policy

All original data including ELISA, Western-blot and RT-PCR are available in Source Data files.

Research involving human participants, their data, or biological material

Policy information about studies with human participants or human data. See also policy information about sex, gender (identity/presentation), and sexual orientation and race, ethnicity and racism.

Reporting on sex and gender	Human head and neck cancer tumor specimens were collected in a de-identified manner without any knowledge of sex/ gender
Reporting on race, ethnicity, or other socially relevant groupings	Human head and neck cancer tumor specimens were collected in a de-identified manner without any knowledge of race/ ethnicity or socially relevant groupings
Population characteristics	not applicable
Recruitment	Patients were consented to the Dana-Farber head and neck cancer tumor banking protocol from our multidisciplinary clinics as they presented for their care.<br><br>All HNSCC patient samples were collected under Dana-Farber/ Harvard Cancer Center institutional review board (IRB) approved protocol #09-472 and studies were performed according to DFCI approved protocol #18-092.<br><br>Peripheral blood mononuclear cells (PBMCs) were isolated using BD Vacutainer® CPT™ (BD Biosciences, # 362760) from apheresis leukoreduction collars which were collected by Crimson Core at Brigham and Women's Hospital as fresh byproducts of platelet donation from anonymized healthy donors (domestic study ID# T0847), under the protocol Mass General Brigham IRB# 2005P001742 and the protocol DFCI-IRB# NHSR#428198.
Ethics oversight	Dana-Farber Cancer Institute institutional review board

Note that full information on the approval of the study protocol must also be provided in the manuscript.

Field-specific reporting

Please select the one below that is the best fit for your research. If you are not sure, read the appropriate sections before making your selection.

- [x] Life sciences
- [ ] Behavioural & social sciences
- [ ] Ecological, evolutionary & environmental sciences

For a reference copy of the document with all sections, see nature.com/documents/nr-reporting-summary-flat.pdf

Life sciences study design

All studies must disclose on these points even when the disclosure is negative.

Sample size	For in vitro experiments, biological replicates (n=3) were included<br>For in vivo mouse experiments, n=5~7 mice per group.
Data exclusions	No data were excluded.
Replication	Two or three independent experiments were conducted in Fig.1c-d, Fig. 3f, Fig.4d-g, and Fig. 7i-j.
Randomization	For in vivo mice experiments, mice were randomized to treatment groups.
Blinding	For in vivo or in vitro studies, authors were not blinded to treatment groups.

Reporting for specific materials, systems and methods

We require information from authors about some types of materials, experimental systems and methods used in many studies. Here, indicate whether each material, system or method listed is relevant to your study. If you are not sure if a list item applies to your research, read the appropriate section before selecting a response.

Materials & experimental systems

n/a	Involved in the study	
	x	Antibodies
	x	Eukaryotic cell lines
x		Palaeontology and archaeology
	x	Animals and other organisms
x		Clinical data
x		Dual use research of concern
x		Plants

Methods

n/a	Involved in the study	
x		ChIP-seq
	x	Flow cytometry
x		MRI-based neuroimaging

Antibodies

Antibodies used	Immunoblotting was performed using the following antibodies: Phospho-STING (Ser366) (Cell Signaling Technology, #50907), STING Cell Signaling Technology, #13647), IRF3 (Cell Signaling Technology, #11904), phospho-IRF3 (Cell Signaling Technology, # 4947), TBK: Cell Signaling Technology, #3504), phospho-TBK1 (Cell Signaling Technology, # 5483), STAT1 (Cell Signaling Technology, #9172) phospho-STAT1 (Cell Signaling Technology, #9167), PTPN2 (Proteintech, # 11214-1-AP), PTPN1 (Abcam,#ab244207), TREX1 (Abcam, #ab185228) and B-Actin (Cell Signaling Technology, #3700). Secondary antibodies were from LICOR Biosciences: IRDye 800CW Goat anti-Rabbit IgG (#926-32211) and IRDye 680LT Goat anti-Mouse IgG (#926-68020). The imaging of blots was acquired using the LICOR Odyssey system.<br><br>For fow cytometric cell viability assay, Annexin V (BioLegend, #640912) and Helix NP™ Green (BioLegend, #425303) were used according to the manufacturer's protocol.<br><br>For profiling tumor-infiltrating immune cells, the following flow cytometry antibodies were used: Live/dead IR (Invitrogen, #L10119), CD45-BUV395 (BD Bioscience, # 564279), TCRB-FITC (BioLegend, #109205), NK1.1-Brilliant Violet 421 (BioLegend, # 156537), CD4-PE/Dazzle™ 594 (BioLegend, #100456), CD8a-Alexa Fluor® 700 (BioLegend, #155022), F4/80-APC (Biolegend, #123116), CD11b-PE (Biolegend, #101208), CD11c-FITC (Biolegend, #117305), CD103-PE/Dazzle™ 594 (Biolegend, #121430), MHC class I| -PerCP/Cyanine5.5 (Biolegend, # 116416).<br>For intracellular staining, Foxp3-APC (Invitrogen, #17-5773-82) and Perforin-PE (BioLegend, #154306) were used.<br><br>For the immunoblotting assay, all primary antibodies were used at a 1:1000 dilution, and secondary antibodies were used at a 1:5000 dilution.<br><br>For immunofluorescence imaging, the primary antibody was used at a 1:200 dilution, and secondary antibodies were used at a 1:1000 dilution.<br><br>For the flow cytometry assay, antibodies were used at a 1:50 dilution.<br><br>For 3D microfluidics killing assay, Hoechst 33342 was used at a 1:800 dilution, and DRAQ7 was used at a 1:100 dilution.
Validation	These antibodies were validated according to the manufacturer's website.

Eukaryotic cell lines

Policy information about cell lines and Sex and Gender in Research

Cell line source(s)	Human HNSCC cell lines BICR-56, CAL33, HSC-2, HSC-4, SCC-4, SCC-9, SCC-25, SNU-899, SNU-1041, UMSAA-14A, and YD8 cell lines, were obtained from Peter Hammerman lab and Thomas E. Carey lab. Lung cancer cell line A549, were obtained from the Broad Institute and authenticated by STRs. THP-1 cells were obtained from ATCC.<br>Mouse HNSCC cell lines MOC1, MOC, and MOC1-esc1 cells were originally generated in our group.
Authentication	HSC-2, SCC-25, SNU-899, YD8 and HeLa were authenticated by Short Tandem Repeats (STRs) genotyping
Mycoplasma contamination	Mycoplasma infection was routinely examined by PCR using the conditioned media after 6 days of culture, using the Mycoplasma Detection kit (ATCC, # 30-1012K).
Commonly misidentified lines<br>(See ICLAC register)	None

Animals and other research organisms

Policy information about studies involving animals; ARRIVE guidelines recommended for reporting animal research, and Sex and Gender in Research

Laboratory animals	6~8 weeks old female C57BL/6 mice or immunodeficient female NOD.Cg-PrkdcscidIl2rgtm1Wjl/SzJ (NSG) mice (Strain # 005557) ordered from the Jackson Laboratory.
Wild animals	None
Reporting on sex	Only female C57BL/6 mice or NOD.Cg-PrkdcscidIl2rgtm1Wjl/SzJ (NSG) mice (Strain # 005557) mice were used.
Field-collected samples	None
Ethics oversight	Research complies with relevant ethical regulations. Specifically, studies using the MOC1-esc1 tumor model were conducted at Broad Institute, under protocol # 0110-08-16-3, approved by the Institutional Animal Care and Use Committee (IACUC) of the Broad Institute.<br><br>Study using the MOC2 tumor model study was conducted at DFCI, under protocol # 04-111, approved by the IACUC of DFCI.

Note that full information on the approval of the study protocol must also be provided in the manuscript.

Plants

Seed stocks

Report on the source of all seed stocks or other plant material used. If applicable, state the seed stock centre and catalogue number. If plant specimens were collected from the field, describe the collection location, date and sampling procedures.

Novel plant genotypes

Describe the methods by which all novel plant genotypes were produced. This includes those generated by transgenic approaches, gene editing, chemical/radiation-based mutagenesis and hybridization. For transgenic lines, describe the transformation method, the number of independent lines analyzed and the generation upon which experiments were performed. For gene-edited lines, describe the editor used, the endogenous sequence targeted for editing, the targeting guide RNA sequence (if applicable) and how the editor was applied.

Authentication

Describe any authentication procedures for each seed stock used or novel genotype generated. Describe any experiments used to assess the effect of a mutation and, where applicable, how potential secondary effects (e.g. second site T-DNA insertions, mosiacism, off-target gene editing) were examined.

Flow Cytometry

Plots

Confirm that:

- [x] The axis labels state the marker and fluorochrome used (e.g. CD4-FITC).
- [x] The axis scales are clearly visible. Include numbers along axes only for bottom left plot of group (a 'group' is an analysis of identical markers).
- [x] All plots are contour plots with outliers or pseudocolor plots.
- [x] A numerical value for number of cells or percentage (with statistics) is provided.

Methodology

Sample preparation

For in vitro cell experiments, cells were collected by 0.25% trypsin, similar to standard tissue culture protocol.
For profiling tumor-infiltrating immune cells, the tumors were dissociated into single cells through the kit from Miltenyi Biotec (#130-096-730) according to the manufacturer's instructions.
For intracellular staining, fixation and permeabilization of cells were performed using the Foxp3 / Transcription Factor Staining Buffer Set (Invitrogen, # 00-5523-00) according to the manufacturer's protocol.

Instrument

For profiling tumor-infiltrating immune cells, CytoFLEX S Flow Cytometer (Beckman Coulter) was used.
For other experiments, cells were acquired and analyzed by the BD LSRFortessa™ Cell Analyzer.

Software

FlowJo (v10)

Cell population abundance

For profiling tumor-infiltrating immune cells, live CD45+ positive cells were above 80% in the MOC1-esc1 tumor on day 12.
For the in vitro cell experiment, pure cell line samples were used.

Gating strategy

Single cells were gated by FSC-H/ FSC-A.
Live CD45+ cells were gated as LIVE/DEAD™ - CD45+ population.
Define distinctive immune cells based on the indicated surface marker.

- [x] Tick this box to confirm that a figure exemplifying the gating strategy is provided in the Supplementary Information.
