# Supplementary material for: PTPN2 inhibition unleashes response to STING agonism in head and neck squamous cell cancer
Source: Nat Commun. 2026 May 2;17:5958. doi: 10.1038/s41467-026-72372-1 (PMC13342608; doi:10.1038/s41467-026-72372-1)
Supplement: Supplementary file 3 — Transparent Peer Review file [file 41467_2026_72372_MOESM3_ESM.pdf]

nature portfolio

Peer Review File

PTPN2 inhibition unleashes response to STING agonism in head and neck squamous cell cancer

Corresponding Author: Professor David Barbie

Parts of this Peer Review File have been redacted as indicated to remove third-party material.

This file contains all reviewer reports in order by version, followed by all author rebuttals in order by version.

Version 0:

Reviewer comments:

Reviewer #1

(Remarks to the Author)
Activation of the cGAS-STING pathway in tumor cells can stimulate cell death and overcome resistance to cancer immunotherapy. However, STING agonists have not been successful in clinical trials. At least one reason for this may be low tumor STING expression. The authors suggest that for STING agonist resistant HNSCC, this may be attributable to the induction of the tyrosine phosphatase PTPN2, a key negative regulator of IFN/JAK/STAT1 signaling. They show that targeting PTPN2 with the dual PTPN1/PTPN1 inhibitor AC484 that is in phase 1 clinical trials can overcome this limitation by 1) recruiting IFNg-producing NK cells and ii) enhancing IFNg-induced STAT1 signaling and thereby STING expressing in tumour cells. Using a syngeneic HNSCC mouse model the authors demonstrate that combined AC484 administration and STING agonism efficiently represses tumor growth and enhances survival. This is an interesting study with important translational implications.

Specific comments:
The study shows that PTPN2 levels are elevated across a range of human HNSCC cells lines and that this correlates with low STING protein levels. Interestingly, (although MW standards for western blots have not been included) it would appear that the 48 kDa endoplasmic reticulum (ER)-targeted variant of PTPN2 (expressed abundantly in human cells), rather than the 45 kDa variant that translocates to the nucleus and dephosphorylates STAT1, is elevated in HNSCC tumor cells. If true, this may be important, since STING is a transmembrane protein on the ER facing the cytosol, as is the case for the 48kDa PTPN2. STING translocates to the Golgi where it recruits TBK1 that phosphorylates STING itself and IRF3. Does the 48 kDa PTPN2 affect STING localisation and phosphorylation? What is the impact of specifically deleting the 48 kDa PTPN2 on STING levels and the IFN/STAT1 response? The authors could use RNAi to specifically target the PTPN2 variants.

The authors take advantage of AC484 to target PTPN2. However, AC484 also inhibits another JAK/STAT phosphatase PTPN1 that like the 48 kDa PTPN2 is also targeted to the ER. Previous studies have shown that the two enzymes can act together to negatively regulate JAK/STAT signaling. How much of the effect on STAT1 tyrosine phosphorylation and STING induction can be attributed to the inhibition of PTPN2 versus PTPN1? The authors could address this by deleting PTPN2 alone, PTP1B alone or both.

The authors use AC484 at 10 microM for in vitro experiments, a concentration that far exceeds its cellular EC50 (176 nM) for the promotion of STAT1 phosphorylation in B16 melanoma cells (Ref 26). At such doses, it is likely that this inhibitor will also inhibit other tyrosine phosphatases. Key experiments (including that described below) should be repeat at lower doses.

The authors demonstrate that AC484 increases NK IFNg production in a NK/HSC-2 co-culture model. This should be repeated with a no HSC-2 tumour cell control to rule out tumour/NK cell crosstalk. This is important as some disagreement exists in the literature as to the function of PTPN2 and PTPN1 in NK cells.

Interestingly the authors show that in vivo AC484 plus ADU enhances the recruitment of NK cells but not T cells to repress tumor growth and prolong mouse survival. What is the reason for the enhanced recruitment of NK cells. Is there a specific chemokine being produced/secreted by AC484/ADU treated tumor cells? Is it the NK cell recruitment, or the depletion of Tregs and activation of CD8+ T cells, that enhances the repression of tumor growth? What is the impact of NK cell depletion

on tumor growth? Can the authors recapitulate these findings using a second syngeneic tumor model.

Minor comments:
The authors should include MW standards and quantify relevant blots including the PTPN2 variants in Fig. 3c.

Reviewer #2

(Remarks to the Author)
The manuscript sumbitted by Li et al. investigates the interconnection between IFNg-STAT1-mediated regulation of STING through a regulatory role of PTPN2/1. Studying HNSCC, the authors demonstrate that a recently developed PRPN2/1 inhibitor (doi.org/10.1038/s41586-023-06575-7) is able to upregulate STING expression in combination with IFNg and they conclude that this is accomplished through STAT1 signaling.

Overall, the manuscript has multiple interesting findings but based on the current quality and amount of data presented in the manuscript I do not find it adequate for publication. I encourage the authors to be more explicit on what the data truly shows and not make overstatements. Furthermore, many data lack significant more controls to support the findings, and finally, the language and textural structure need to be improved.

Major points to consider

1. The authors use ADU-S100 throughout the whole study. This is a major limitation as this compound is shown to be very unstable and poor cell penetrating. Many of the arguments made throughout the manuscript regarding cancer cells lines not responding to STING activation (See Fig. 1A-E, 2B-E, 4B-G, 6D-I) could merely be that ADU-S100 is not taken up by the cells. Therefore, the authors should also test either HT-DNA (delivered by lipofectamine) or a small molecule agonist such as diABZI, which has been proven to easily penetrate cell membranes.
2. An example of inappropriate statement and lack of clarity can be seen on line 88. Here, the authors state that HNSCC tumor cells lines express low STING and make a clear reference. However, that reference only mentioned the MOC1 and MOC2 (which was developed in the lab of one of the senior authors on this manuscript). And even so, in the referenced manuscript MOC1 and MOC2 are shown to have low STING expression when compared to immune cells, which does not equal generally low expression. Thus, such generic statements do not hold true. Also, the reference 24 clearly shows that multiple HPV negative HNSCC tumors do express STING so the statement on line 94 “regardless of HPV status” is not correct.
3. Regarding point 2 – other groups have shown that MOC2 for example has STING expression and do respond to DNA and RT-induced activation, which contradict multiple statements made in this manuscript. See doi:10.1016/j.xcrm.2024.101528. It would be appropriate for the authors to address this. Also, the authors themselves do show in extended data fig 3 that both MOC1, MOC2 and MOC1-esc1 do express STING. Why was MOC2 and MOC1-esc1 not tested with IFNb stimulation? And why not include a relevant ISG as control for the assay?
4. Throughout the manuscript the quality of WB is not acceptable. In Fig 1a there is a clear “white band” on the p-IRF3 staining suggesting a burned signal on the membrane. The authors need to either explain this or better yet redo the staining. There also needs to be better comparison with loading controls. Often there are very large differences as to how much protein is loaded. It is very difficult to understand the rationale for comparing all the HNSCC data with THP1 cells – completely other origin and importantly THP1 cells are also a cancer cell line and not just a “monocytic cell”. Utilizing the HNSCC FaDu cell line, which expresses responsive STING, might be a better alternative.
5. Fig 1b should include a positive control for activation such as PolyI:C
6. Figure 1 panel b, c,d,e and h are examples where the transparency level of data analysis could be improved. The authors need to show individual dot plots. This also applies with many of the other figures. For example, in figure 3e the authors shows error bars on a single measuring time but not on the rest. This is unacceptable practice and puts into question the correctness of data. Furthermore, in figure 2 panel c, d and e, there are multiple cases where only one dot per patient is presented but then later three dots are shown in another panel. This is not general practice. Also, statistical analysis are made on many figures where there are no error bars presented (figure 3e, 3g, 4g, 5c). This seems odd.
7. Statements made based on no statistical significance. Throughout the manuscript, conclusions are made based on data which show no statistical significance (see Fig 1c-d, 2c-d, f, 4c-e.g etc.). One example can be seen on line 159, in which the authors state “IFN-b was also significantly increased upon ADU treatment..” referencing figure 2e, However, figure 2d looking at TNF-a, CCL5, and CXCL10 show no significant differences. Therefore it is misleading to state “IFN-b was also..”.
8. Inconsistency between data is an issue. As an example, figure 1b and 1h both show HSC-2 cells stimulated with ADU-S100. In 1b there is no increased CXCL10 level but in 1h there actually is, with no significance marker in 1h.
9. Data not supporting statement. In line 269 the authors argues that “Without concurrent ADU treatment, AC484 and IFN-γ increased STING protein levels did not promote STING activation on its own, as measured by p-IRF3 induction (Fig. 4b)”. To make such statement the authors should at minimum do a phosphor S366 STING western blot. Also pIRF3 can be achieved by many other pathways independent of STING.
10. The data presented in figure 6 is much more valid and is currently the most supportive information related to the manuscript. However, it would be essential to repeat such data, so it is not the result of a single in vivo study done with 5-7 mice. Also, in figure 6d there seems to be no rationale for what CD45+ cells are depleted to allow elevated NK cells in the treated groups (compare overall % freq between 6d and 6e). Another important experiment to conduct is to test AC484 and ADU-S100 in STING deficient mouse to show if the antitumoral effect is host or cancer cell dependent.
11. For figure 6 it would be essential to show an updated western blot analysis of all factors related to the cGAS-STING pathway +/- AC484, ADU-S100, which will confirm the basal level of protein expression.
12. Many of the statements in the discussion need to be softened.

13. The methods section pertaining to the in vivo studies are severely lacking to ensure reproducibility. At minimum the journal guidelines should be followed.

Minor
1. CD45neg cells are not tumor cells but could be fibroblasts, endothelia and epithelial cells. See line 169
2. The authors should highlight that drugs like DNMT and EZH2 are very unspecific drugs and are known to be cytotoxic. See line 196-198.
3. Regarding the statement on line 333, it would be good for the authors to explain the discrepancy between MOC1 and MOC1-esc1 regarding aPD-1 resistency, as it has been shown that MOC1 tumors do respond to aPD1 – see PMC5712281 – and that the MOC1-esc1 is a single clone of escape as reported in doi.org/10.1136/jitc-2021-004034. Maybe the authors should also use the VAF/SNV analysis from this paper to explore cGAS-STING pathway expression
4. Extended figure 4 – how would the authors explain TBK1 degradation after ADU-S100 treatment. Data suggest that cell death may be part of reason why protein bands disappear after numerous hours.
5. Extended figure 7. The CD45 plot looks very weird. There are multiple populations suggesting that the antibody has not been titrated correctly. Also where are the FMO to support the gating strategy? These analyses need to be remade and do question the correctness of the analysis done in figure 6.
6. Language consistency – Throughout the manuscript different language is used to describe the same thing. As an example see line 33 “Tumor cell STING”, vs. line 99 “Cancer cell STING”.
7. Line 119: The authors write that p-IRF3 is not induced despite STING turnover, however this may be a kinetic issue rather than no induction.
8. Line 120: Change upregulated to increased as phosphorylations are not an upregulation.
9. Line 139: The authors state that they have overexpressed STING in these HSC-2 cells, however how was this overexpression achieved.
10. Line 142: To broad of a statement
11. Line 199: “Thus generated TREX1 versus control knockout HSC-2 cells”. Unclear what this means.
12. Line 113-115: The use of the word “upregulated” is misused as the data being referred to shows the baseline protein levels and not an upregulation.
13. Line 238: p-STAT1 is observed in the generated STAT1 KO cell line upon treatment with IFN-g and AC484 Fig. 3f.
14. Line 253-254: Misleading statement. “p-STAT1 and STING levels were consistently induced… further conforming the robustness of this result”. However, one of the included cell lines, SNU-1041 does not show convincing STING induction.
15. Line 273: The authors reference STING phosphorylation. This needs to be confirmed with a p-STING specific antibody.
16. Line 192: The authors conclude that “CXCL10 and IFN-b production from AC484/IFNg treated HSC-2 cells are in an ADU dose-dependent manner”. However, the data only shows that IFN-b and not CXCL10 increases in a dose-dependent manner.
17. It would be interesting to see if AC484 treatment has an effect on the author’s tumor explant models, as these also contain native NK cells.
18. Immunehistochemsitry: It would be nice to see other IHC markers included in figure 6b such as CD45, EpCAM, and other downstream factors for STING activation.
19. Line 346: The authors mention no change in macrophage levels after treatment. However, in figure 7d a significant decrease is shown.

Reviewer #3

(Remarks to the Author)
This manuscript presents a compelling investigation into the role of PTPN2 in suppressing STING expression in head and neck squamous cell carcinoma (HNSCC) and proposes PTPN2/1 inhibition (via AC484) as a strategy to restore STING expression and sensitize tumors to STING agonists like ADU-S100. The authors demonstrate that PTPN2 suppresses STAT1 activity, preventing IFNγ-mediated STING induction, thereby contributing to tumor resistance to STING-based therapies. By combining AC484 with IFNγ, the study shows STING expression is restored, leading to enhanced response to STING agonism in vitro and in vivo, with a notable role for NK cells in the tumor microenvironment (TME). This work is novel because the authors showed that PTPN2 is a suppressor of STING expression in HNSCC. The study fills an important gap by proposing PTPN2 as a previously unrecognized mechanism of tumor-intrinsic STING suppression. They demonstrate that PTPN2 negatively regulates STAT1 activation, limiting STING expression and immune response in tumors. The study suggests that PTPN2 inhibition could improve the efficacy of STING agonists, offering a new immunotherapeutic combination for PD-1-resistant HNSCC.
The study clearly defines the molecular mechanism by which PTPN2 suppresses STING via STAT1 repression. It provides direct evidence that AC484 restores STING expression and sensitizes tumors to STING agonists. The authors used HNSCC cell lines, patient tumor explants, and an anti-PD-1-resistant mouse model to validate findings. They show restoration of STING expression, cytokine secretion, and tumor regression after AC484 and ADU treatment. The study highlights PTPN2 as a targetable checkpoint that could improve STING-based immunotherapies. The findings support further clinical testing of AC484/STING agonist combinations in HNSCC and potentially other cancers.

This is a high-quality manuscript that identifies PTPN2 as an immune checkpoint suppressing STING in HNSCC and provides a rational strategy for overcoming resistance to STING agonists. However, the study would be significantly strengthened by additional mechanistic experiments to:
1. Confirm the direct role of PTPN2 in suppressing STING. Please support it by genetic modification and not using inhibitors.
2. Differentiate the contributions of NK vs. CD8+ T cells in the AC484/ADU response, and their source of IFNg.

3. What would be the efficacy of this treatment combination in cold tumors like the MOC2 tumors?

Reviewer #4

(Remarks to the Author)
I co-reviewed this manuscript with one of the reviewers who provided the listed reports. This is part of the Nature Communications initiative to facilitate training in peer review and to provide appropriate recognition for Early Career Researchers who co-review manuscripts.

Version 1:

Reviewer comments:

Reviewer #1

(Remarks to the Author)
The authors have addressed my key concerns

Reviewer #2

(Remarks to the Author)
The authors have made substantial revision that stregthen the overall quality of the manuscript and the findings.

However, I still find it inadequate that the authors have not included a statement regarding the limitation of only using ADU-S100 as a direct STING agonist, knowning the issues there has been reported with this drug and its poor pharmacological effects. This should be mentioned in the discussion limitation section.

Reviewer #3

(Remarks to the Author)
I appriciate the effort, and I support the publication of this paper

Open Access This Peer Review File is licensed under a Creative Commons Attribution 4.0 International License, which permits use, sharing, adaptation, distribution and reproduction in any medium or format, as long as you give appropriate credit to the original author(s) and the source, provide a link to the Creative Commons license, and indicate if changes were made.
In cases where reviewers are anonymous, credit should be given to 'Anonymous Referee' and the source.
The images or other third party material in this Peer Review File are included in the article's Creative Commons license, unless indicated otherwise in a credit line to the material. If material is not included in the article's Creative Commons license and your intended use is not permitted by statutory regulation or exceeds the permitted use, you will need to obtain

permission directly from the copyright holder.
To view a copy of this license, visit https://creativecommons.org/licenses/by/4.0/

REVIEWER COMMENTS

Reviewer #1 (Remarks to the Author): with expertise in PTPNs, cancer immunology

Activation of the cGAS-STING pathway in tumor cells can stimulate cell death and overcome resistance to cancer immunotherapy. However, STING agonists have not been successful in clinical trials. At least one reason for this may be low tumor STING expression. The authors suggest that for STING agonist resistant HNSCC, this may be attributable to the induction of the tyrosine phosphatase PTPN2, a key negative regulator of IFN/JAK/STAT1 signaling. They show that targeting PTPN2 with the dual PTPN2/PTPN1 inhibitor AC484 that is in phase 1 clinical trials can overcome this limitation by 1) recruiting IFNg-producing NK cells and ii) enhancing IFNg-induced STAT1 signaling and thereby STING expressing in tumour cells. Using a syngeneic HNSCC mouse model the authors demonstrate that combined AC484 administration and STING agonism efficiently represses tumor growth and enhances survival. This is an interesting study with important translational implications.

We thank the reviewer for highlighting the interesting nature of our study and its translational implications, and for their specific comments below that have strengthened the work further.

Specific comments:

1. The study shows that PTPN2 levels are elevated across a range of human HNSCC cells lines and that this correlates with low STING protein levels. Interestingly, although MW standards for western blots have not been included) it would appear that the 48 kDa endoplasmic reticulum (ER)-targeted variant of PTPN2 (expressed abundantly in human cells), rather than the 45 kDa variant that translocates to the nucleus and dephosphorylates STAT1, is elevated in HNSCC tumor cells. If true, this may be important, since STING is a transmembrane protein on the ER facing the cytosol, as is the case for the 48kDa PTPN2. STING translocates to the Golgi where it recruits TBK1 that phosphorylates STING itself and IRF3. Does the 48 kDa PTPN2 affect STING localization and phosphorylation? What is the impact of specifically deleting the 48 kDa PTPN2 on STING levels and the IFN/STAT1 response? The authors could use RNAi to specifically target the PTPN2 variants.

This is an interesting point, as to whether the 48kDa PTPN2 isoform may regulate STING directly; however, inhibition of PTPN2 does not appreciably alter the timing of STING phosphorylation or translocation-mediated degradation (**Revised Fig.4b, Supplementary Fig.4**). Of note, the PTPN2 antibody we have been utilizing from Proteintech (#11214-1-AP) has been knockout validated (See Tian Xia, et al, PNAS. 2019. Fig. 2A). Moreover, using A549 cells that specifically express the 48 kDa band (See Stéphanie Bussières-Marmen, et al, Methods. 2014), we confirm that deleting PTPN2 genetically primarily removes this dominant 48 kDa band (**Revised Supplementary Fig 3b-c**). We also confirm that specific deletion of this PTPN2 band unleashes IFN/STAT1 and STING expression, in contrast to deletion of PTPN1 which has a minor effect (**Revised Supplementary Fig. 3b-e**). We further confirm that the impact of PTPN2 knockout on STING induction occurs at the mRNA level (**Revised Supplementary Fig. 3g**), in consonance with our findings using the small molecule inhibitor. Mechanistically, the TC48 isoform of PTPN2 has been shown to mediate the inhibition of STAT1 phosphorylation (Maria Kleppe, et al., Haematologica. 2011) and may do this indirectly by dephosphorylating JAK kinases as they associate with cytokine receptors during their transit through the ER-Golgi system to the plasma membrane. We plan to investigate the isoform-specific roles of TC45 and TC48 in dampening IFN sensitivity and STING expression in future studies.

2.The authors take advantage of AC484 to target PTPN2. However, AC484 also inhibits another JAK/STAT phosphatase PTPN1 that like the 48 kDa PTPN2 is also targeted to the ER. Previous studies have shown that the two enzymes can act together to negatively regulate JAK/STAT signaling. How much of the effect on STAT1 tyrosine phosphorylation and STING induction can be attributed to the inhibition of PTPN2 versus PTPN1? The authors could address this by deleting PTPN2 alone, PTP1B alone or both.

As above, we deleted PTPN2, PTPN1, or both and find that PTPN2 knockout has a more substantial impact as compared with PTPN1 knockout on inducing pSTAT1 and STING levels in response to IFNg treatment. We find that double knockout can slightly further enhance pSTAT1 induction, but this does not additively increase STING levels further (**Revised Supplementary Fig. 3b-e**).

3.The authors use AC484 at 10 microM for in vitro experiments, a concentration that far exceeds its cellular EC50 (176 nM) for the promotion of STAT1 phosphorylation in B16 melanoma cells (Ref 26; Baumgartner, C. K. et al. *Nature*, 2023). At such doses, it is likely that this inhibitor will also inhibit other tyrosine phosphatases. Key experiments (including that described below) should be repeat at lower doses.

This is another important point - in the Baumgartner manuscript that described this inhibitor (led by one of our co-senior authors Robert Manguso together with Abbvie), 10 μM of AC484 was utilized across multiple experiments in order to achieve >IC 90 inhibition and completely ablate signaling (Baumgartner, C. K. et al. *Nature*, 2023). Moreover, in this prior work PTPN9 is the only other phosphatase that showed inhibition but at much higher concentrations and no other phosphatases were found in off target profiling even at much higher concentrations. Multiple experiments were also previously conducted confirming that doses of 10 μM on cells phenocopies the dual KO of PTPN2/PTPN1. Moreover, in some assays concentrations above 10 μM were needed to see the maximal effect that is achievable by dual deletion of PTPN2/PTPN1.

To address this question in the HNSCC context, we now also compare genetic PTPN2 knockout with dose dependent response of AC484, finding that maximal STAT1 mediated induction of STING occurs at 10 μM, matching the same impact of PTPN2 knockout and substantiating the specific role of PTPN2 in this phenotype (**Revised Supplementary Fig. 3f, g**).

4.The authors demonstrate that AC484 increases NK IFNg production in a NK/HSC-2 co-culture model. This should be repeated with a no HSC-2 tumour cell control to rule out tumour/NK cell crosstalk. This is important as some disagreement exists in the literature as to the function of PTPN2 and PTPN1 in NK cells.

We also thank the reviewer for this excellent point and now confirm that NK cells alone treated with AC484 can produce IFNγ (**Revised Supplementary Fig. 6a**). This is consistent with prior work showing AC484 targets both tumor cells and immune cells including T and NK cells (Baumgartner, C. K. et al. *Nature*, 2023).

5. Interestingly, the authors show that in vivo AC484 plus ADU enhances the recruitment of NK cells but not T cells to repress tumor growth and prolong mouse survival. What is the reason for the enhanced recruitment of NK cells. Is there a specific chemokine being produced/secreted by AC484/ADU treated tumor cells?

AC484/ADU induced STING signaling and downstream IFN activation results in production of key NK cell chemo-attractants including CXCL10/CCL5 production, which we demonstrate (**Revised Fig.2c,e; Fig.4c; Fig. 5j**) and are known to mediate tumor cell STING induced NK cell migration (Erik H Knelson, et al. Cancer Immunology Research 2022; Tetsuo Tani, et al. Cancer Discovery 2024). Indeed, we further validated NK cell migration in conjunction with production of these chemokines using a similar 3D microfluidic model system *ex vivo*. NK cells loaded in the left side channel of the 3D microfluidic device significantly migrate to the central channel and kill HSC-2 tumor spheroids (**Revised Fig. 5k-m**). While both CCL5 and CXCL10 are known to recruit activated T cells as well, the mechanism explaining the absence of T cell recruitment is currently unknown and an active area of investigation for our labs. Prior evidence suggests that STING activation may have direct toxicity to T cells (Erik H Knelson, et al., Cancer Immunol Res. 2022, Bridget Larkin, et al., J Immunol. 2017, Muhammet F Gulen, et al., Nat Commun. 2017, Silvia Cerboni, et al., J Exp Med. 2017), and we are investigating this possibility.

Is it the NK cell recruitment, or the depletion of Tregs and activation of CD8+ T cells, that enhances the repression of tumor growth? What is the impact of NK cell depletion on tumor growth? Can the authors recapitulate these findings using a second syngeneic tumor model.

These are excellent points and we therefore performed multiple *in vivo* experiments to address these comments. First, we compared response of MOC1-esc1 tumors to AC484/ADU in syngeneic vs NSG mice, which confirms that response is dependent on T or NK cells due to near complete loss of activity in NSG mice (**Revised Fig.7h**). In contrast, depletion of NK cells alone was not able to block efficacy, suggesting multifactorial involvement of immune cells in vivo, potentially related to the Treg depletion that the reviewer highlights (**Revised Fig 7i, j**). To this end, we also attempted CD8+ T cell depletion studies. While we did not observe a reversal of the efficacy, we also unfortunately observed incomplete T cell depletion, so it is not possible to draw confident conclusions from this study, and we have therefore not included it.

Lastly, per the reviewer's suggestion we also recapitulated the effects of AC484/ADU in the additional HNSCC syngeneic MOC2 tumor model (**Revised Fig. 6g**).

Minor comments:
The authors should include MW standards and quantify relevant blots including the PTPN2 variants in Fig. 3c.

Thanks for the suggestion. We added the MW standards for the PTPN2 blot in which we performed CRISPR-Cas9 knockout, proving specificity for the 48KDa band (**Revised Supplementary Fig. 3b,c**). We have added this language to the text and now also clarify in **Fig.3c** that the dominant band is the 48KDa isoform.

Reviewer #2 (Remarks to the Author): with expertise in STING, cancer immunology

The manuscript submitted by Li et al. investigates the interconnection between IFNg-STAT1-mediated regulation of STING through a regulatory role of PTPN2/1. Studying HNSCC, the authors demonstrate that a recently developed PRPN2/1 inhibitor (doi.org/10.1038/s41586-023-06575-7) is able to upregulate STING expression in combination with IFNg and they conclude that this is accomplished through STAT1 signaling.

Overall, the manuscript has multiple interesting findings but based on the current quality and amount of data presented in the manuscript I do not find it adequate for publication. I encourage the authors to be more explicit on what the data truly shows and not make overstatements. Furthermore, many data lack significant more controls to support the findings, and finally, the language and textural structure need to be improved.

We also very much appreciate this reviewer's overall positive comment in addition to the constructive criticism, which has substantially improved the manuscript as we detail below. In addition to addressing their important points experimentally, we have also removed overstatements and improved the clarity.

Major points to consider

1. The authors use ADU-S100 throughout the whole study. This is a major limitation as this compound is shown to be very unstable and poor cell penetrating. Many of the arguments made throughout the manuscript regarding cancer cells lines not responding to STING activation (See Fig. 1A-E, 2B-E, 4B-G, 6D-I) could merely be that ADU-S100 is not taken up by the cells. Therefore, the authors should also test either HT-DNA (delivered by lipofectamine) or a small molecule agonist such as diABZI, which has been proven to easily penetrate cell membranes.

We completely agree with the reviewer about these relative limitations of ADU-S100 (despite it being more ENPP1 resistant than 2'-3' cGAMP). We have shown previously that at higher concentrations as utilized in this study it can indeed be taken up by cancer cells (Kitajima, et al, Cancer Cell 2022) and activate tumor cell STING in mesothelioma patient derived explant models that retain STING expression (Knelson et al., Cancer Immunol Res 2022). To address this critique, alongside HSC-2 cells we have now included a different positive control cancer cell line (H196 cells) which respond robustly to ADU-S100 (Mahadevan et al., Cancer Discovery 2021), and HeLa cells as a well established negative control cell line that suppresses STING signaling via HPV18 E7 (Lau et al., Science 2015). These data demonstrate that ADU can activate STING signaling in H196 cells, but not HSC-2 cells or HeLa cells (**Revised Fig.1a, b**). In addition, overexpression of STING in HSC-2 cells increased ADU sensitivity, which we point out further confirms that ADU is still capable of membrane permeability in HSC-2 cells at these same concentrations (**Revised Fig.1g, h**). Finally, IFNg/PTPN2i induced STING expression restores ADU-S100 sensitivity in HSC-2 cells, as measured by increases in downstream p-TBK1, p-IRF3 (**Fig.4b**), IFNb production (**Fig.4d**), as well as cytotoxicity (**Fig. 4e-g**), arguing that cell penetration is not the issue, but rather the issue is lack of baseline STING expression.

We also understand that it is important to strengthen the findings by utilizing an independent method of activating cGAS-STING signaling, and thus now demonstrate that transfection with poly(dG:dC) (dsDNA mimic) also efficiently induces IFNb production in HSC-2 cells pre-treated with AC484/IFNg (**Revised Supplementary Fig. 1a, 5a**).

We thank the reviewer for these comments which has improved robustness.

2. An example of inappropriate statement and lack of clarity can be seen on line 88. Here, the authors state that HNSCC tumor cells lines express low STING and make a clear reference (Ellen Moore, et al., *Cancer Immunology Research*, 2016). However, that reference only mentioned the MOC1 and MOC2 (which was developed in the lab of one of the senior authors on this manuscript). And even so, in the referenced manuscript MOC1 and MOC2 are shown to have low STING expression when compared to immune cells, which does not equal generally low expression. Thus, such generic statements do not hold true.
Also, the reference 24 (Saulters, E. et al. *Cancer Research Communications*, 2023) clearly shows that multiple HPV negative HNSCC tumors do express STING so the statement on line 94 "regardless of HPV status" is not correct.

We thank the reviewer for allowing us to improve clarity and avoid generic statements, as they are correct that especially the levels of mRNA expression (despite being low) are being shown relative to immune cell populations. We have clarified this in the text and removed the comment "regardless of HPV status".

See below for the revised introductory paragraph (**Revised lines 92-105**).

"HNSCC patients with low STING expression is associated with shorter progression-free survival with standard of care therapy16. Furthermore, mouse oral squamous cell carcinoma (MOC1) and MOC2 cells exhibited low STING expression relative to multiple immune cell populations and exhibited impaired response to CDNs4, 12, 23. Prior work also suggested that CDN-induced TNF-α and IFN-β were mainly derived from myeloid cell populations in syngeneic mouse tumors4, 12, though systematic comparison of STING levels or responsiveness in HNSCC relative to other tumor cell lines was not performed. Recent work has also confirmed suppression of STING expression in multiple human HNSCC cell lines, as well as lack of response to the STING agonist ADU-S100 as compared with THP1 myeloid cells24. This result was similar in HPV positive cells as well as HPV negative cells regardless of STING levels. However, once again, direct comparison with other known STING agonist responsive human cancer cell lines was not performed, and the underlying mechanism that mediates suppression of STING signaling in HNSCC cells has been relatively underexplored."

3. Regarding point 2 – other groups have shown that MOC2 for example has STING expression and do respond to DNA and RT-induced activation, which contradict multiple statements made in this manuscript. See doi:10.1016/j.xcrm.2024.101528. It would be appropriate for the authors to address this. Also, the authors themselves do show in extended data fig 3 that both MOC1, MOC2 and MOC1-esc1 do express STING. Why was MOC2 and MOC1-esc1 not tested with IFNb stimulation? And why not include a relevant ISG as control for the assay?

We thank the reviewer for highlighting this reference from the Barber laboratory (Delaunay et al., Cell Rep Med 2024), which is actually consistent with our findings and shows that the endogenous STING expression in MOC2 cell is low (Fig 7A from this manuscript is shown below). Robust activation of STING signaling by IR requires co-delivery of their "Nano-STAVs" and incubation with CD11c+ DCs with MOC2 cells (Fig 7I, J from that paper). Moreover, work from other groups (Ellen Moore, et al., *Cancer Immunology Research*, 2016) also showed that MOC2 cells express low STING.

[editorial note: third party material redacted]

Consistent with these published results, we also show that the basal level of STING in MOC1 and MOC2 cell is low. But AC484/IFNg treatment can efficiently increase STING (**Revised Supplementary Fig. 3h, i**).

We observed that AC484 plus IFNg, but not IFNb, increased STING in HSC-2 cells and MOC1 cells (**Revised Supplementary Fig. 3a, h**), thus we focused on AC484/IFNg but not AC484/IFNb on additional tumor models including MOC2 and MOC1-esc1(**Revised Supplementary Fig. 3i, j**). In addition, AC484/IFNg increased STING restored ADU response, evidence by increased IFNb production upon ADU treatment in both MOC1 and MOC2 model (**Revised Supplementary Fig. 5c, d**).

4. Throughout the manuscript the quality of WB is not acceptable. In Fig 1a there is a clear “white band” on the p-IRF3 staining suggesting a burned signal on the membrane. The authors need to either explain this or better yet redo the staining. There also needs to be better comparison with loading controls. Often there are very large differences as to how much protein is loaded. It is very difficult to understand the rationale for comparing all the HNSCC data with THP1 cells – completely other origin and importantly THP1 cells are also a cancer cell line and not just a “monocytic cell”. Utilizing the HNSCC FaDu cell line, which expresses responsive STING, might be a better alternative.

We apologize for this blot that was over-exposed. To address the important point 1 raised above, we have repeated the blot in comparison with other cancer cell lines at multiple timepoints, without over-exposure. As above, we used H196 as a known robust positive control cell line for ADU responsiveness, and HeLa cells as a known negative control (**Fig.1a**).

5. Fig 1b should include a positive control for activation such as PolyI:C.

We appreciate this excellent suggestion as well. We directly compared transfection with poly(dG:dC) (dsDNA mimic) with transfection with Poly(I:C) (dsRNA mimic) for 24 hours in HSC2 cells, and find that only Poly(I:C) substantially increases IFNb production (**Revised Supplementary Fig. 1a**).

6. Figure 1 panel b, c,d,e and h are examples where the transparency level of data analysis could be improved. The authors need to show individual dot plots. This also applies with many of the

other figures. For example, in figure 3e the authors show error bars on a single measuring time but not on the rest. This is unacceptable practice and puts into question the correctness of data. Furthermore, in figure 2 panel c, d and e, there are multiple cases where only one dot per patient is presented but then later three dots are shown in another panel. This is not general practice. Also, statistical analysis are made on many figures where there are no error bars presented (figure 3e, 3g, 4g, 5c). This seems odd.

We apologize for not including dot plots originally and have now addressed statistical concerns throughout the manuscript.

With respect to Figure 2c, this relates to the nature of the Luminex assays performed on conditioned media from many of the tumor explant samples, which are incredibly expensive and usually conducted for screening. This was also due to limited patient material from some patients, such that we were not able to perform duplicates and triplicates all of the time. To address this important point and generate statistical significance we have now pooled the ADU vs control conditions for all samples, which clearly demonstrates that TNF-α, CCL5, CXCL10, and IFN-beta met statistical significance as being significantly induced across the cohort.

7. Statements made based on no statistical significance. Throughout the manuscript, conclusions are made based on data which show no statistical significance (see Fig 1c-d, 2c-d, f, 4c-e.g etc.). One example can be seen on line 159 (**Revised Line 173**), in which the authors state "IFN-b was also significantly increased upon ADU treatment.." referencing figure 2e (**Revised Fig.2c**), However, figure 2d (**Revised Fig.2c**) looking at TNF-a, CCL5, and CXCL10 show no significant differences. Therefore it is misleading to state "IFN-b was also..".

As above we have now addressed these concerns throughout and apologize for the prior lack of clarity.

8. Inconsistency between data is an issue. As an example, figure 1b and 1h both show HSC-2 cells stimulated with ADU-S100. In 1b there is no increased CXCL10 level but in 1h there actually is, with no significance marker in 1h.

The difference between these figures is that HSC-2 cells in 1h were transfected with a control DNA plasmid, to account for any contribution of STING overexpression to the DNA plasmid itself. These data show that there is a slight effect of the plasmid on CXCL10 levels, which is a very sensitive marker, but this is markedly amplified by STING over-expression.

9. Data not supporting statement. In line 269 (**Revised Line 293**) the authors argues that "Without concurrent ADU treatment, AC484 and IFN-γ increased STING protein levels did not promote STING activation on its own, as measured by p-IRF3 induction (Fig. 4b)". To make such statement the authors should at minimum do a phosphor S366 STING western blot. Also pIRF3 can be achieved by many other pathways independent of STING.

We also appreciate this point and have now included phospho serine 366 STING levels (p-STING) in 4b, which is corroborates our statement that induction of STING protein was not associated with STING activation since p-STING was negative.

10. The data presented in figure 6 is much more valid and is currently the most supportive information related to the manuscript. However, it would be essential to repeat such data, so it is not the result of a single in vivo study done with 5-7 mice. Also, in figure 6d (**Revised Fig.7a**) there seems to be no rationale for what CD45+ cells are depleted to allow elevated NK cells in

the treated groups (compare overall % freq between 6d and 6e) (**Revised Fig.7a and 7b**). Another important experiment to conduct is to test AC484 and ADU-S100 in STING deficient mouse to show if the antitumoral effect is host or cancer cell dependent.

We also thank the reviewer for these important points to improve robustness. We validated that AC484/ADU treatment strongly suppresses MOC1-esc1 tumor in vivo in additional two independent experiments (**Revised Fig. 7h-j**). And further repeated these findings in the separate MOC2 model (**Revised Fig. 6g**).

We also find that macrophages in CD45+ cells are decreased in AC484/ADU group (**Revised Supplementary Fig. 7c**), potentially contributing to the increase in NK cells. Moreover, as pointed out by reviewer #1, FOXP3+ Tregs are also decreased, though the total CD4 count is unaffected (**Revised Fig. 7b, f**).

We also agree that it is important to assess tumor cell dependency of the phenomenon. We have addressed this comment in 2 different ways. First, we compared response in syngeneic mice with NSG immunodeficient mice, to determine whether the effect is host immune cell dependent versus tumor cell dependent (**Revised Fig. 7h**). To address the specific STING dependence of the phenomenon, we also utilized our 3D *ex vivo* human modeling system, having needed to conduct 4 additional *in vivo* experiments to address other reviewer concerns and due to the general guidance from NIH to expand utilization of human models relative to mouse models. To test whether restoration of tumor cell intrinsic STING is directly responsible for this effect, we knocked out STING in HSC-2 cells using CRISPR/Cas9 (**Revised Fig. 5i**) and loaded spheroids into collagen for co-culture with NK cells in a microfluidic device. In contrast to control HSC-2 spheroids, STING-defective HSC-2 tumor spheroids were resistant to NK cell migration and killing following AC484/ADU treatment (**Revised Fig. 5k-m**).

11. For figure 6 it would be essential to show an updated western blot analysis of all factors related to the cGAS-STING pathway +/- AC484, ADU-S100, which will confirm the basal level of protein expression.

We also appreciate this point, but the restoration of STING is tumor cell specific, as evidenced by immunohistochemistry (IHC) data which shows uniquely increased expression in tumor cells, and not stroma in the AC484 group. We have also now added p-TBK1 in the AC484/ADU group, which is strongly induced, and coincides with evidence of STING turnover in tumor cells treated with the combination therapy (**Revised Figs 6b, c**). As whole tumor tissue consists of admixed tumor cells, immune cells (T cells, macrophages, NK cells), fibroblasts, endothelial cells, pericytes, and other tissue-resident cells that express STING, it is not possible to discriminate this effect using whole tumor lysates.

12. Many of the statements in the discussion need to be softened.

We agree with this point and have substantially toned down the discussion, including a limitation of the study section as well (**Revised Line 494**).

13. The methods section pertaining to the in vivo studies are severely lacking to ensure reproducibility. At minimum the journal guidelines should be followed.

Thanks for the comments. We have improved the methods by including more details.

Minor
1. CD45neg cells are not tumor cells but could be fibroblasts, endothelia and epithelial cells. See line 169 (**Revised Line 184**).

We have revised the statement, "ADU treatment failed to induce CXCL10 from $CD45^{-}$ cells, but did induce response from CD45 positive immune cells (**Fig. 2e**)" Please see **Line 180**.

2. The authors should highlight that drugs like DNMT and EZH2 are very unspecific drugs and are known to be cytotoxic. See line 196-198 (**Revised Line 211-213**).

Thanks for the comments. We used a relatively low concentration of the DNMT inhibitor (decitabine; 100 nM) and the EZH2 inhibitor (Tazemetostat; 5 μM), which showed low cytotoxicity but increased STING in the A549 (positive control. See Shunsuke Kitajima., Cancer Discovery. 2019). However we have also added a comment that these drugs can be cytotoxic and that we cannot fully rule out an impact of epigenetic silencing (**Revised Line 111-112**).

3. Regarding the statement on line 333, it would be good for the authors to explain the discrepancy between MOC1 and MOC1-esc1 regarding aPD-1 resistance, as it has been shown that MOC1 tumors do respond to aPD1 – see PMC5712281 – and that the MOC1-esc1 is a single clone of escape as reported in doi.org/10.1136/jitc-2021-004034. Maybe the authors should also use the VAF/SNV analysis from this paper to explore cGAS-STING pathway expression.

This is a good suggestion. Previous work from our group (doi.org/10.1136/jitc-2021-004034. Liye Zhou, et al., *Journal for ImmunoTherapy of Cancer*. 2022) reported that MOC1-esc1 tumors are highly infiltrated by Tregs and M2-like tumor-associated macrophages, which result in anti-PD1 resistance. We have added a sentence to explain this in the results section, and also highlight that combination therapy decreases both of these populations in our flow cytometry data (see response to main point #10) (See **Revised Fig. 7f and Supplementary Fig. 7c**). We also tested STING protein levels in both MOC1 and MOC1-esc1 by western-blot. They both express low STING, but AC484/IFNg treatment can increase STING, suggesting that PTPN2 mediated STING repression is a feature of both models (**Revised Supplementary Fig. 3h-i**).

4. Extended figure 4 – how would the authors explain TBK1 degradation after ADU-S100 treatment. Data suggest that cell death may be part of reason why protein bands disappear after numerous hours.

When activated TBK1, like STING, trafficks to the autophagosome and is turned over by lysosomal autophagy, see previous work from our group (Yang, et al. Cancer Immunology Research. 2016) and others (Xueying Wang, et al. Theranostics. 2025). Typically this is the active p-TBK1 fraction that undergoes lysosomal degradation, accounting for a subfraction of total TBK1.

5. Extended figure 7. The CD45 plot looks very weird. There are multiple populations suggesting that the antibody has not been titrated correctly. Also where are the FMO to support the gating strategy? These analyses need to be remade and do question the correctness of the analysis done in figure 6.

Thanks for the comments. We have re-gated the CD45 population and including all the CD45 positive cells. In addition, we re-quantified the all the immune cells in revised **Fig.7** and **Supplementary Fig. 7.**
See below.

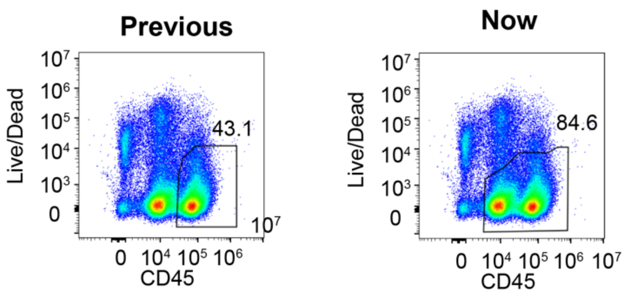

6. Language consistency – Throughout the manuscript different language is used to describe the same thing. As an example see line 33 “Tumor cell STING”, vs. line 99 “Cancer cell STING”.

We thank the reviewer for this comment to improve language consistence. We now use “Tumor cell STING” only.

7. Line 119: The authors write that p-IRF3 is not induced despite STING turnover, however this may be a kinetic issue rather than no induction.

This is a good point. We now compare p-IRF3 levels at both 4 hours and 24 hours which demonstrates the increase at 4 hours but decrease at 24 hours, consistent with the negative feedback regulation described above due to lysosomal autophagy (**Revised Fig.1a**).

8. Line 120: Change upregulated to increased as phosphorylations are not an upregulation.

We have also addressed the point and use “increased” only.

9. Line 139: The authors state that they have overexpressed STING in these HSC-2 cells, however how was this overexpression achieved.

We apologize and have added the information in the methods – we used a lentiviral expression construct along with a control vector (**See Line 639**).

10. Line 142: To broad of a statement
Taken together, these data confirm that HNSCC cell lines in general express low levels of STING and are refractory to STING agonists, but remain sensitive to STING agonist response when STING expression is restored.

We have toned down this description (**Revised Line 158**).
“Taken together, these data confirm that HNSCC cell lines generally express low levels of STING but are potentially able to respond to STING agonists when STING expression is elevated.”

11. Line 199 (**Revised Line 214**): “Thus generated TREX1 versus control knockout HSC-2 cells”. Unclear what this means.

We revised the description to explain this more clearly, “thus generated TREX1 knockout HSC-2 cells by CRISPR-Cas9.”

The scramble sgRNA indicated the non-targeting sgRNA from the Get-go library v2. sgRNAs target human TREX1 were cloned into the lentiCRISPRv2 puro plasmid (**Revised Line 635**).

12. Line 113-115: The use of the word “upregulated” is misused as the data being referred to shows the baseline protein levels and not an upregulation.

As above we appreciate this point and now use the word “increased” instead.

13. Line 238: p-STAT1 is observed in the generated STAT1 KO cell line upon treatment with IFN-g and AC484 Fig. 3f.

We conducted second independent experiments but weak p-STAT1 band is still observed, likely reflecting the fact that we could not achieve 100% knockout. The other possible reasons may include:
1) Alternative Splicing: The STAT1 knockout might have affected only one isoform of STAT1, while another remains present and phosphorylated.
2) Antibody Non-Specificity/Cross-Reactivity: The anti-p-STAT1 antibody we used might be binding to other phosphorylated proteins of similar molecular weight, such as phosphorylated STAT3 (86 KDa) or p-STAT5 (90 KDa). The molecular weight of p-STAT1 is 91 KDa.

14. Line 253-254 (**Revised Line 275-277**): Misleading statement. “p-STAT1 and STING levels were consistently induced… further conforming the robustness of this result”. However, one of the included cell lines, SNU-1041 does not show convincing STING induction.

Thanks for the comments. We appreciate this point, but compared with basal STING levels in this cell line, AC484/IFNg treatment still exhibits a strong fold change in STING upregulation in SNU-1041 cell (**Fig.3j**). We have toned this language down, adding the point that SNU-1041 did show an increase in STING, but that the total levels were still modest.

See **Revised Line 275-278**:

“p-STAT1 and STING levels were consistently induced by combination treatment across HNSCC cell lines examined, further confirming the generalizability of this result (**Fig. 3j**). Notably, SNU-1041 showed an increase in STING, but the total levels remained modest.”

15. Line 273: The authors reference STING phosphorylation. This needs to be confirmed with a p-STING specific antibody.

We have now added the p-STING blot in **Fig. 4b**.

16. Line 292 (**Revised Line 314**): The authors conclude that “CXCL10 and IFN-b production from AC484/IFNg treated HSC-2 cells are in an ADU dose-dependent manner”. However, the data only shows that IFN-b and not CXCL10 increases in a dose-dependent manner.

We apologize for this mistake and have revised the statement.
See **Revised Line 316:**

“ADU treatment strongly promoted CXCL10 and IFN-β production from AC484/IFN-γ treated HSC-2 cells, using both low and high ADU concentrations (10 μM vs 50 μM) (**Fig. 4c,d**).”

17. It would be interesting to see if AC484 treatment has an effect on the author’s tumor explant models, as these also contain native NK cells.

This is a great suggestion and will be analyzed in subsequent work, given the broad existing scope of the current manuscript.

18. Immunohistochemistry: It would be nice to see other IHC markers included in figure 6b such as CD45, EpCAM, and other downstream factors for STING activation.

Thanks for the suggestion. As above, we have now added p-TBK1 IHC results (**Revised Fig. 6b, c**), which was significantly increased in the AC484/ADU treated group. In addition, CD45 had been analyzed by flow cytometry (**Revised Fig. 7a; Supplementary Fig. 7a**).

19. Line 346 (**Revised Line 409**): The authors mention no change in macrophage levels after treatment. However, in Extended figure 7d a significant decrease is shown.

Above we have now revised the description to state the decrease in macrophage levels (**Revised Supplementary Fig. 7c**).

Reviewer #3 (Remarks to the Author): with expertise in HNSCC

This manuscript presents a compelling investigation into the role of PTPN2 in suppressing STING expression in head and neck squamous cell carcinoma (HNSCC) and proposes PTPN2/1 inhibition (via AC484) as a strategy to restore STING expression and sensitize tumors to STING agonists like ADU-S100. The authors demonstrate that PTPN2 suppresses STAT1 activity, preventing IFNγ-mediated STING induction, thereby contributing to tumor resistance to STING-based therapies. By combining AC484 with IFNγ, the study shows STING expression is restored, leading to enhanced response to STING agonism in vitro and in vivo, with a notable role for NK cells in the tumor microenvironment (TME). This work is novel because the authors showed that PTPN2 is a suppressor of STING expression in HNSCC. The study fills an important gap by proposing PTPN2 as a previously unrecognized mechanism of tumor-intrinsic STING suppression. They demonstrate that PTPN2 negatively regulates STAT1 activation, limiting STING expression and immune response in tumors. The study suggests that PTPN2 inhibition could improve the efficacy of STING agonists, offering a new immunotherapeutic combination for PD-1-resistant HNSCC.

The study clearly defines the molecular mechanism by which PTPN2 suppresses STING via STAT1 repression. It provides direct evidence that AC484 restores STING expression and sensitizes tumors to STING agonists. The authors used HNSCC cell lines, patient tumor explants, and an anti-PD-1-resistant mouse model to validate findings. They show restoration of STING expression, cytokine secretion, and tumor regression after AC484 and ADU treatment. The study highlights PTPN2 as a targetable checkpoint that could improve STING-based immunotherapies. The findings support further clinical testing of AC484/STING agonist combinations in HNSCC and potentially other cancers.

This is a high-quality manuscript that identifies PTPN2 as an immune checkpoint suppressing STING in HNSCC and provides a rational strategy for overcoming resistance to STING agonists. However, the study would be significantly strengthened by additional mechanistic experiments to:

1. Confirm the direct role of PTPN2 in suppressing STING. Please support it by genetic modification and not using inhibitors.

Thanks for the suggestion. We generated PTPN2 and PTPN1 knockout HSC-2 cells and found that PTPN2, instead of PTPN1, plays the major role in suppressing IFN/STAT1 mediated STING induction (**Revised Supplementary Fig. 3b-e, g**).

2. Differentiate the contributions of NK vs. CD8+ T cells in the AC484/ADU response, and their source of IFNg.

In response to this and other reviewers, we have performed multiple *in vivo* experiments to address these comments. First, we compared response of MOC1-esc1 tumors to AC484/ADU in syngeneic vs NSG mice, which confirms that response is T or NK cell dependent due to near complete loss of activity in NSG mice (**Revised Fig.7h**). This is consistent with our *in vitro* findings that immune cell infiltration and IFNg in general is needed for AC484 to prime this response.

We successfully depleted NK cells alone, but this was not able to block efficacy, suggesting multifactorial involvement of immune cells *in vivo*, (**Revised Fig 7i, j**). We also attempted CD8+ T cells depletion as well and did not block efficacy, but unfortunately T cell depletion was incomplete so it is difficult to draw firm conclusions from this study and we have therefore not

included it. As reviewer #1 also highlights, these results may be related to the significant Treg depletion that we also observe, and is difficult to control for with pure NK and CD8+ T cell depletion.

Because it is difficult to measure IFN-γ production in vivo, we have instead focused on perforin positive NK cells as an alternative approach, which is known to correlate with IFN-γ production (**Revised Fig.7c-e**). We thus can clearly demonstrate that activated NK cells are infiltrate into the TME in response to the combination, though highlight in the discussion that we can isolate a specific role for NK cells alone in this phenotype and that future work is needed to dissect the contribution of Treg depletion and other possible immune cell types.

3. What would be the efficacy of this treatment combination in cold tumors like the MOC2 tumors?

We thank the reviewer for this important comment. We have now validated that AC484/ADU treatment also efficiently suppresses MOC2 tumor growth *in vivo* (**Revised Fig. 6g**). In detail, 1/5 mice from AC484 and ADU monotherapy group exhibited durable tumor control, but 4/5 mice from AC484/ADU combination therapy showed persistent tumor control (**Revised Fig. 6g**).

Reviewer #4 (Remarks to the Author):

I co-reviewed this manuscript with one of the reviewers who provided the listed reports. This is part of the Nature Communications initiative to facilitate training in peer review and to provide appropriate recognition for Early Career Researchers who co-review manuscripts.

REVIEWER COMMENTS

Reviewer #1 (Remarks to the Author):

The authors have addressed my key concerns.

We thank the reviewer.

Reviewer #2 (Remarks to the Author):

The authors have made substantial revision that stregthen the overall quality of the manuscript and the findings.

However, I still find it inadequate that the authors have not included a statement regarding the limitation of only using ADU-S100 as a direct STING agonist, knowning the issues there has been reported with this drug and its poor pharmacological effects. This should be mentioned in the discussion limitation section.

We thank the reviewer for this excellent point. We have added the limitation of ADU-S100 in the discussion limitation section.

“Finally, ADU-S100 has important pharmacologic limitations as a first-generation STING agonist. Thus, next generation systemic STING agonists, including novel formulations as payloads on tumor cell targeting antibody drug conjugates (Fahey CG, et al., *Cancer Cell*, 2026), have substantially greater therapeutic potential to combine with PTPN2/1 inhibitors in the clinic.”

Reviewer #3 (Remarks to the Author):

I appreciate the effort, and I support the publication of this paper.

We thank the reviewer.
